# Vestiges of an Ancient Border in the Contemporary Genetic Diversity of North-Eastern Europe

**DOI:** 10.1371/journal.pone.0130331

**Published:** 2015-07-01

**Authors:** Anu M. Neuvonen, Mikko Putkonen, Sanni Översti, Tarja Sundell, Päivi Onkamo, Antti Sajantila, Jukka U. Palo

**Affiliations:** 1 Laboratory of Forensic Biology, Department of Forensic Medicine, PO Box 40 (Kytösuontie 11) FI-00014, University of Helsinki, Helsinki, Finland; 2 Department of Biology, Section of Genetics and Physiology, University of Turku, Itäinen Pitkäkatu 4, 20014, Turku, Finland; 3 Department of Biosciences, PO Box 56 (Viikinkaari 5) FI-00014, University of Helsinki, Helsinki, Finland; 4 Department of Philosophy, History, Culture and Art Studies, PO Box 59 (Unioninkatu 38 F) FI-00014, University of Helsinki, Helsinki, Finland; 5 Institute of Applied Genetics, Department of Molecular and Medical Genetics, University of North Texas Health Science Center, 3500 Camp Bowie Blvd, Fort Worth, Texas, 76107, United States of America; IPATIMUP (Institute of Molecular Pathology and Immunology of the University of Porto), PORTUGAL

## Abstract

It has previously been demonstrated that the advance of the Neolithic Revolution from the Near East through Europe was decelerated in the northernmost confines of the continent, possibly as a result of space and resource competition with lingering Mesolithic populations. Finland was among the last domains to adopt a farming lifestyle, and is characterized by substructuring in the form of a distinct genetic border dividing the northeastern and southwestern regions of the country. To explore the origins of this divergence, the geographical patterns of mitochondrial and Y-chromosomal haplogroups of Neolithic and Mesolithic ancestry were assessed in Finnish populations. The distribution of these uniparental markers revealed a northeastern bias for hunter-gatherer haplogroups, while haplogroups associated with the farming lifestyle clustered in the southwest. In addition, a correlation could be observed between more ancient mitochondrial haplogroup age and eastern concentration. These results coupled with prior archeological evidence suggest the genetic northeast/southwest division observed in contemporary Finland represents an ancient vestigial border between Mesolithic and Neolithic populations undetectable in most other regions of Europe.

## Introduction

In Europe, human history has been decisively shaped by two events: the colonization of Europe by modern humans c. 45 000 years ago (45 kya) and the spread of agricultural technology from the Fertile Crescent in Asia Minor to north of Europe 9–5 kya. The latter Neolithisation process has been a major transformation period in the history of most European populations [1–4].

Over the years, there has been much controversy regarding the mechanism by which the agricultural lifestyle advanced from the Near East to western and northern Europe (see [5] and references therein). According to the current view, genetic evidence appears to favor intermediate scenarios between the opposing cultural diffusion model (advance of technology) and the demic diffusion model (advance of Neolithic people) [6]. After colonizing the Balkans, the Neolithic advance continued westwards by two main routes, along the Mediterranean coast and through the Central European plains following the Danube [4, 7]. Eventually, this led to the admixture of Mesolithic hunter-gatherer and Neolithic farmer gene pools (see e.g. [8]). Studies based on analyses of contemporary genetic diversity have estimated widely varying proportions for genes traceable to the Paleolithic and Neolithic gene pools [9–11]. This picture has been further elucidated by ancient DNA (aDNA) studies that have helped to identify Mesolithic (hunter-gatherer) and Neolithic (farmers) genetic elements [6, 12–14]. Recently, ancient DNA and isotope analyses have shown that in Central Europe the two livelihoods, and also gene pools, existed in parallel for extended periods after the Neolithic influx of people and technology [15].

The contribution of Neolithic genes in the present European gene pool dominate, but in various degrees, in different parts of Europe [6]. Although variation between different ancient or contemporary DNA data sets can be partly explained by differences in quantity and in interpretive methodology (see [9, 16]), there appears to be regional variation that probably traces back to the local Neolithisation processes. In this context, the events in the western and northern extremes of Europe may have been totally different compared to the Central Europe (*cf*. [7]). Archaeological studies based on radiocarbon dating show that when reaching northern parts of Europe, the speed of the Neolithic advance has slowed down. Although this might be explained by the time required for development of crops adapted to northern latitudes, Isern et al. (2012) [17] suggested, based on simulation studies, that this has mainly been due to niche occupation by a sizeable population of Mesolithic people who based their subsistence on foraging. Competition on resources has reduced Neolithic population growth and delayed the advance and admixture [18]. If this hypothesis holds, it would entail that the Mesolithic and Neolithic genetic signatures could be more discernible in the marginal regions of Europe, as suggested by [7].

Finland has been a region that was among the last to adopt the sedentary agricultural way of life in Europe, perhaps even as late as 500 BC [19] although the issue is contentious (see e.g. [20]). Intriguingly, a genetic border separating South-Western and Eastern Finland has been identified in a number of human genetic studies, especially in the male-mediated Y-chromosomes [21–23]. This is rather exceptional, when contrasted against the largely clinal variation elsewhere in Europe [24]. The border localized by genes also appears to coincide with a medieval political border and, more importantly, with a border in many cultural features—but not with any apparent geographical borders [23].

Here, we present and explore a hypothesis that the geographic patterns actually denote a still existing border—genetic and cultural—between Neolithic farmers and Mesolithic hunter-gatherers. We focus on the haplogroup-level diversity of mtDNA and Y-chromosomes in Finland today. Obviously, ancient DNA data would hold a capacity for more direct analysis of the proposed scenario, but samples for these analyses are unavailable. Local conditions rarely allow survival of ancient biological samples over a thousand years, and one is forced to focus on contemporary genetic patterns.

## Materials and Methods

**Fig 1:**
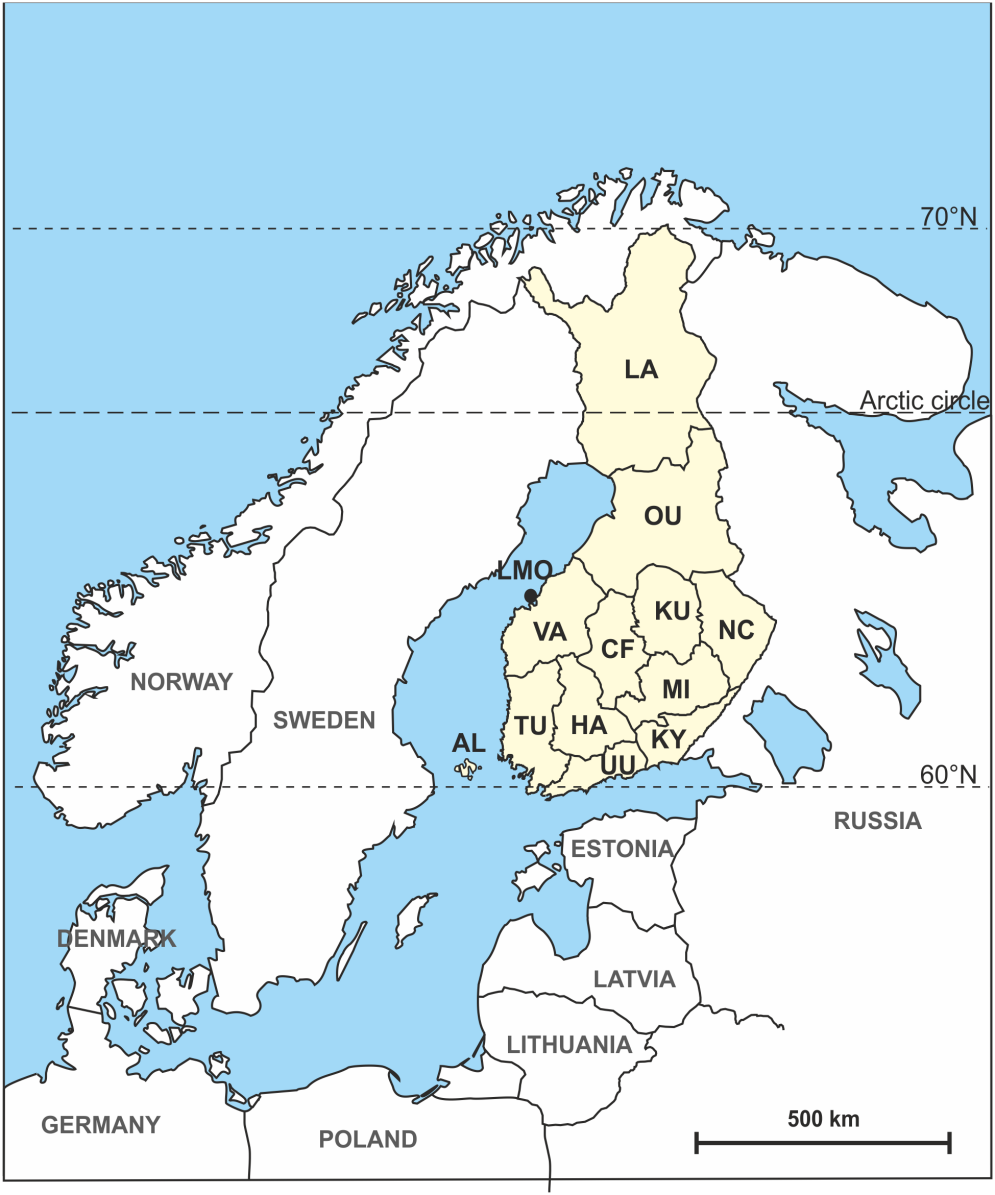
Map of Northern Europe with Finland and the different subpopulations analyzed.

The data comprises mitochondrial DNA and Y-chromosomal data, with the main focus on haplogroup distribution and diversity in different parts of Finland. The samples were collected from voluntary donors with written informed consents, limiting use of the samples to the characterization of geographical patterns of neutral genetic variation in Finland. The consent forms were signed upon sampling and included information on study design and an option to decline the use of samples at any time in the future. It also specified publication of the results in anonymized form in scientific series (ethics committee approval: Helsinki University Central Hospital’s Ethical Committee Dnro 329/13/03/00/2013). The samples were assigned based on donors’ place of residence to 13 different subpopulations (Fig 1). For both mtDNA and Y-chromosomal (below) data basic diversity indices were estimated using Arlequin v. 3.5.1.3 [25]. Haplogroup frequencies, number of haplotypes (*A*) and haplotype diversity (*Ĥ*) were estimated for different haplogroups on the different geographic levels (all samples, regions and subpopulations). Differentiation was assessed by estimating pairwise *F*_ST_ and *Φ*_ST_ indices. The statistical dispersion values associated with the diversity indices was determined through 10 000 randomization steps.

### Mitochondrial DNA data

Two different Finnish mitochondrial DNA sequence data sets were analyzed in this study:

1. Hypervariable regions 1 and 2 data (HVR1+2; *N* = 832), consisting of 643 base pairs (aligned length; sites 16024–16385 and 73–340) defining 384 unique haplotypes. The data was obtained from Palo et al. 2009 [23].
2. Complete mitochondrial sequences from Finland (*N* = 367) were obtained through GenBank searches (*N* = 274, the majority of these are published in [26, 27]) and from the 1000Genomes-project (*N* = 93; [28]). The accession numbers, references and associated information are listed in S1 Table.

The mtDNA haplogroup information was inferred from sequence data using mtDNA tree Build 15 (PhyloTree.org, 30 Sep 2012). For each haplotype, the haplogroup definition was projected with haplogrep [29] [30]. For the HVR1+2 sequences, the phylogenetic sense of the inferred haplogroups was also assessed by inspecting the haplotype position in a phylogenetic tree by eye. For this end, unrooted maximum likelihood (ML) trees were constructed using MEGA v. 5.05 [31] assuming Tamura-Nei+Γ substitution model with shape parameter α = 0.7. As this merely aimed at checking the overall topology, the fit of the mutation model parameters were not formally tested, but the robustness of the topology was checked by constructing the trees also assuming a simpler Jukes-Cantor model.

The HVR1+2 mtDNA data was subdivided into two haplogroup clusters based on their inferred association with 1) Mesolithic hunter-gatherers (HUNT) including haplogroups U and V, and 2) Neolithic farmers (FARM) including haplogroups H, J, T and K. This clustering was based on results from [3]. A conservative assignment strategy was adopted, and all sequences that could not be assigned unambiguously were excluded (see Results).

For the HVR1+2 data haplotype and haplogroup frequencies as well as basic diversity indices (number of haplotypes *A*, haplotype diversity *Ĥ* and nucleotide diversity π) were estimated within haplogroups and within haplogroup clusters HUNT and FARM.

Geographical differences in HUNT and FARM haplogroup frequencies within Finland were visualized by extrapolated maps constructed with MapInfo, and the patterns were tested by linear regression assuming a generalized linear model (GLM). Here, logistic regression analysis in R v.2.14.1, assuming binomial distribution and logit link function, was used to find a bipartition that minimized the product of the *P*-values of combined Hg-frequency differences of HUNT and FARM clusters. As a control for the validity of the partition, the results were contrasted with random non-continuous bipartitions.

In order to explore the demographic dynamics within the HUNT and FARM haplogroup clusters in Finland, complete genome mtDNA data was analyzed to infer past changes in (female) effective population sizes in haplogroups H (*N* = 94) and U (*N* = 86). These groups were chosen as they are considered to represent the hunter-gatherers (U) and farmers (H) well and because similar analyses in these groups have recently been published [32].

Bayesian Skyline Plots (BSPs) were constructed with the BEAST v. 1.7.4 software package programs [33]. The method uses Bayesian coalescence inference from sequence data and Markov Chain Monte Carlo (MCMC) sampling to produce posterior probability distributions for effective population sizes. In order to better fit the mutation model used in the genealogy building, only the coding region (bases 577–16023) sequence data was used in the analyses.

BEAUTi was used to generate input files for the BEAST runs. Six mutation models were fitted to the data, General Time Reversible (GTR) and Hasegawa-Kishino-Yano (HKY; [34]) with three alternative parameter combinations (with proportion of invariants *pinv*, gamma distributed substitutions Γ, and both). The strongest support was obtained for GTR with proportion of invariants set to *pinv* = 0.80, base frequencies estimated from the data, and a lognormal relaxed clock. A clock rate 1.69 x 10^-8^ substitutions^-1^ site^-1^ year^-1^[32] was assumed in the analyses.

For each sequence group, three independent MCMC chains were run for 40 or 80 million steps after a burn-in period with length of 10% of the actual run. Values were recorded every 10,000 steps. These parameters were chosen based on a number of trial runs.

Logcombiner was used to combine the results from multiple runs and the results examined using tracer v. 1.5 [35]. Proper mixing and convergence of runs were confirmed by evaluating effective sampling sizes (ESS) reported for the different parameters as well as by comparing results from independent runs. The ESS values denote the number of independent samples accepted, and ESS > 200 was used as a cut-off for acceptance.

To determine if the BSP model was the best method for reconstructing past population sizes it was compared to the constant population model using Bayes Factors calculated from the marginal likelihoods for each model, as implemented in TRACER. Strength for the BSP model was determined using guidelines on Bayes Factors provided in [36].

### Y-chromosomal DNA data

As with the mtDNA, the Y-chromosomal data set combined Y-STR haplotype (17-locus AmpFlSTR Yfiler) and haplogroup data (N = 584) from two different sources:

1. In-house data set (*N* = 330). For this data, sample collection, DNA extraction and Y-STR typing was performed as described in Palo et al. 2009 [23]. Haplogroup information was obtained through SNP typing (see below) with emphasis on the two main haplogroups in Finland, haplogroups N1c1 and I1 and their subhaplogroups. These two haplogroups represent approximately 90% of Finnish Y-chromosomes.
2. Y-chromosomal data obtained by data mining on the Family Tree website (data mining 12.9.2012, http://www.familytreedna.com/; *N* = 254). This data included 16 out of the 17 Y-STR loci in the AmpFlSTR Yfiler set (barring DYS635) as well as the haplogroup designation defined by SNP-information.

For the Y-chromosomal haplogroup definitions, we follow the International Society of Genetic Genealogy (ISOGG; http://www.isogg.org) nomenclature published in 2015. For the in-house data set, the samples were first preclassified as belonging to haplogroup N1c1, I1 or other based on the Y-STR information using the Haplogroup Predictor algorithm (http://www.hprg.com/hapest5/) with “Northwest Europe” as a metapopulation prior. Haplogroup N-predicted samples were genotyped for M46, M178 and L550, and haplogroup I1 samples were further genotyped for SNPs L22, L258 and L300. Some samples were also designated through comparison of haplotypes with previously typed samples (N1c1 and I1 subhaplogroups).

Real Time PCR genotyping of SNPs M46 was performed using TaqMan technology. Taqman SNP Genotyping Master Mix, with one custom-ordered Genotyping Assay (rs34442126) including sequence-specific primers from Life Technologies (Carlsbad, CA, USA), was used to set up 13 μl reactions including SNP Assay with 5.625 μl of template DNA. Reactions were analyzed on ABI 7500 RT-PCR machine using cycling conditions consisting of a 10-min activation at 95°C, followed by 40 cycles of denaturing at 95°C for 15 s, and extension at 60°C for 1 min.

SNPs M178, L550, L22, L258, L300 were sequenced, with amplification conducted using 1x PCR buffer II (Life Technologies), 1.5 mM MgCl2 (Promega, Madison WI, USA), 200 μM dNTPs (Biofellows Oy via Oligomer, Helsinki, Finland), 2.5 U AmpliTaq Gold polymerase (Life Technologies), 6.5 μg bovine serum albumin (Thermo Fisher Scientific, Waltham, MA, USA), and 0.2 μM of each primer. Approximately 10 ng of genomic template DNA was added to the master mix in each reaction. Cycling conditions consisted of a 7-minute denaturation step at 95°C, followed by annealing and extension at 56°C and 68°C respectively for 33 cycles. For SNP M178 a lower annealing temperature of 51.7°C was used. Amplified fragments were purified enzymatically and sequenced using the PCR primers and BigDye Terminator v1.1 chemistry (Life Technologies). The sequencing reactions were purified using XTerminator Purification Kit (Life Technologies) and analysed on ABI Prism Genetic Analyzer 3130xl. Data were compiled using Sequencher v.4.10 software (GeneCodes Inc., Ann Arbor, MI, USA).

## Results

### mtDNA: HVR1 and HVR2

The HVR1+2 data set consisted of 832 sequences and 384 unique haplotypes, showing an overall haplotype diversity of *Ĥ* = 0.993±0.001. The haplogroup frequencies, deduced from the sequence data, are presented in in Table 1. The overall haplogroup distribution in Finland was similar than in Western Europe, with H as a dominant haplogroup but also with relatively high occurrence of >20% for haplogroup U, and especially U5.

**Table 1:** MtDNA haplogroup frequencies and basic diversity indices.

		FINLAND					SOUTHWEST					NORTHEAST				
Hg		*N*	*f*	*A*	*Ĥ*	*π*	*N*	*f*	*A*	*Ĥ*	*π*	*N*	*f*	*A*	*Ĥ*	*π*
**HUNT**	U	202	0.243	85	0.957 ±0.011	0.010±0.005	70	0.180	42	0.968 ±0.010	0.009 ±0.005	132	0.298	60	0.949 ±0.011	0.010 ±0.005
	V	30	0.036	14	0.830 ±0.063	0.004 ±0.003	12	0.031	10	0.955 ±0.057	0.006 ±0.004	18	0.041	7	0.726 ±0.096	0.003 ±0.002
	**U+V**	**232**	**0.279**	**99**	**0.965 ±0.006**	**0.011 ±0.006**	**82**	**0.211**	**52**	**0.976 ±0.008**	**0.011 ±0.006**	**150**	**0.339**	**67**	**0.957 ±0.009**	**0.011 ±0.006**
**FARM**	H	276	0.332	117	0.976 ±0.004	0.023 ±0.013	133	0.342	71	0.978 ±0.005	0.007 ±0.004	143	0.323	68	0.971 ±0.006	0.007 ±0.004
	J	46	0.055	27	0.941 ±0.024	0.009 ±0.005	33	0.085	22	0.911 ±0.044	0.009 ±0.005	13	0.029	9	0.936 ±0.051	0.010 ±0.006
	T	51	0.061	33	0.977 ±0.009	0.010 ±0.005	28	0.072	21	0.976 ±0.016	0.009 ±0.003	23	0.052	18	0.976 ±0.020	0.011 ±0.006
	K	46	0.055	22	0.917 ±0.027	0.004 ±0.003	24	0.062	16	0.931 ±0.039	0.004 ±0.003	22	0.050	10	0.883 ±0.041	0.004 ±0.003
	**H+J+T+K**	**419**	**0.504**	**199**	**0.987 ±0.002**	**0.011 ±0.006**	**218**	**0.560**	**130**	**0.989 ±0.002**	**0.011 ±0.006**	**201**	**0.454**	**105**	**0.983 ±0.003**	**0.011 ±0.006**
***ALL***	*-*	*832*	*1*.*000*	*384*	*0*.*993 ±0*.*001*	*0*.*012 ±0*.*006*	*389*	*0*.*468*	*236*	*0*.*994 ±0*.*001*	*0*.*013 ±0*.*007*	*443*	*0*.*532*	*225*	*0*.*990 ±0*.*001*	*0*.*012 ±0*.*006*

Hg = haplogroup, *N* = number of samples, *f* = haplogroup frequency, *A* = number of unique haplotypes, *Ĥ* = within-Hg haplotype diversity, π = within-Hg nucleotide diversity.

Out of the 832 haplotypes, 232 (27.9%) and 419 (50.4%) fell in the hunter-gatherer (HUNT; Hgs U, V) and farmer (FARM; H, T, K, J) groups, respectively. The remaining 173 samples represented haplogroups D, HV, I, N, R, W, X and Z.

Results from the GLM regression analysis showed greatest HUNT/FARM frequency differences between southern/western subpopulations AL, TU, HA, VA, UU, LMO and northern/eastern subpopulations MI, CF, KU, KY, NC, OU and LA (lowest product of *p*-values *P*_HUNT_ * P_FARM_ = 9.85E-08). This bipartition was several orders better than for any of the ten random non-continuous bipartitions tested (*P*_HUNT_ * P_FARM_ = 0.002…0.313), suggesting validity.

**Fig 2:**
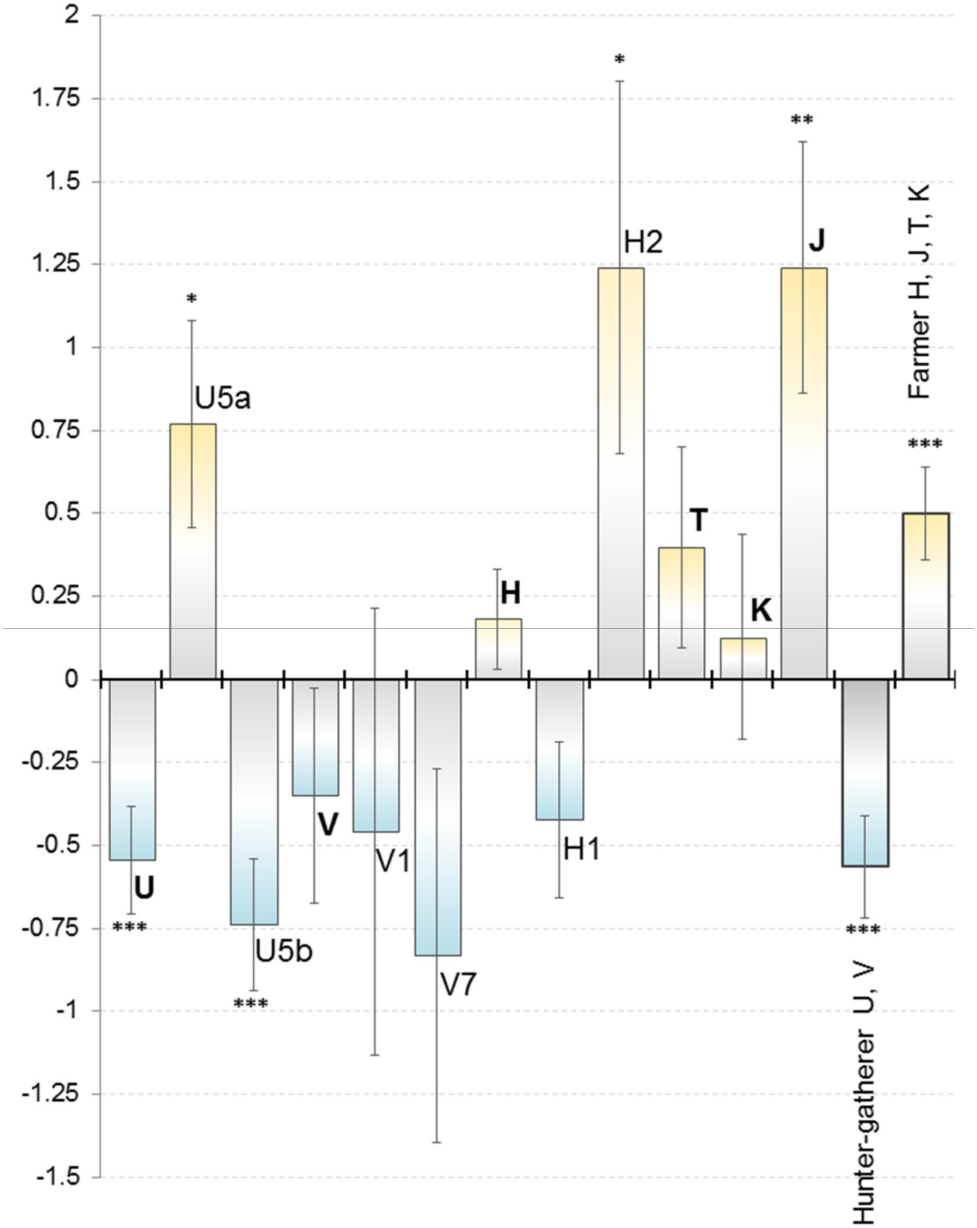
Logistic regression estimates representing the difference in haplogroup frequencies between the SW & NE subpopulations. Above X-axis: SW dominance, below: NE dominance. The results are shown for division (cf. Fig 2) that maximized the difference. Error bars denote standard deviation, statistical significance is marked with stars. No statistically significant values were obtained in randomized, non-continuous divisions.

In the HUNT group, logistic regression estimate for U was -0.54, i.e. showing 54% lower occurrence in SW compared NE (*p* = 0.0008; Fig 2). However, in the subhaplogroup level, the pattern gets more diverse. The overall NE affinity for U can be attributed to strong eastern bias in subhaplogroup U5b, especially U5b1. This subhaplogroup shows clearly higher frequency in Finland than in most other European populations. However, haplogroup U5a shows a lower frequency but also contrasting geographical pattern. Haplogroup V and both its main subhaplogroups show eastern bias.

In the FARM group, the most significant geographical disparity was observed in haplogroup J (164% more in SW, *p* = 1.05E-03). All the other main FARM haplogroups show minor SW bias, but with more fragmented subhaplogroup patterns. Haplogroups H1a and H1f show relatively strong NE bias, but H2 a western bias.

**Fig 3:**
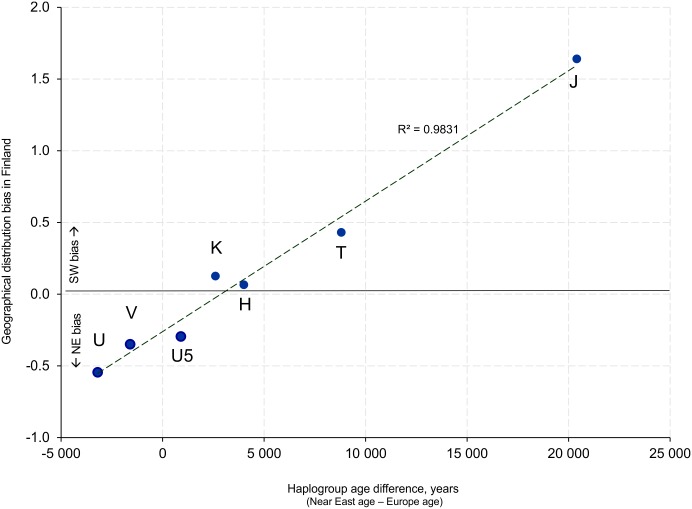
The strength of NE bias for the main haplogroups plotted against the difference of haplogroup ages in Near Eastern and European populations estimated in (Richards, et al. 2000). Haplogroups with older estimated ages in Europe than in Near East show stronger eastern bias in Finland, and vice versa.

Interestingly, the strength of haplogroups NE bias in Finland correlates with the difference of haplogroup ages in Near Eastern and European populations estimated in [37]. Haplogroups with older estimated ages in Europe than in Near East show stronger eastern bias in Finland, and vice versa (Fig 3), with a clear-cut overall correlation R^2^ = 0.983. Spearman’s rank correlation gives the same signal, with *r*_S_ = 0.9643 (*p* < 0.01).

### mtDNA: complete genomes

Altogether 367 complete mitochondrial genomes were obtained from the Genbank and 1000Genomes project. This data showed in general similar overall haplogroup distribution than the control region data.

**Fig 4:**
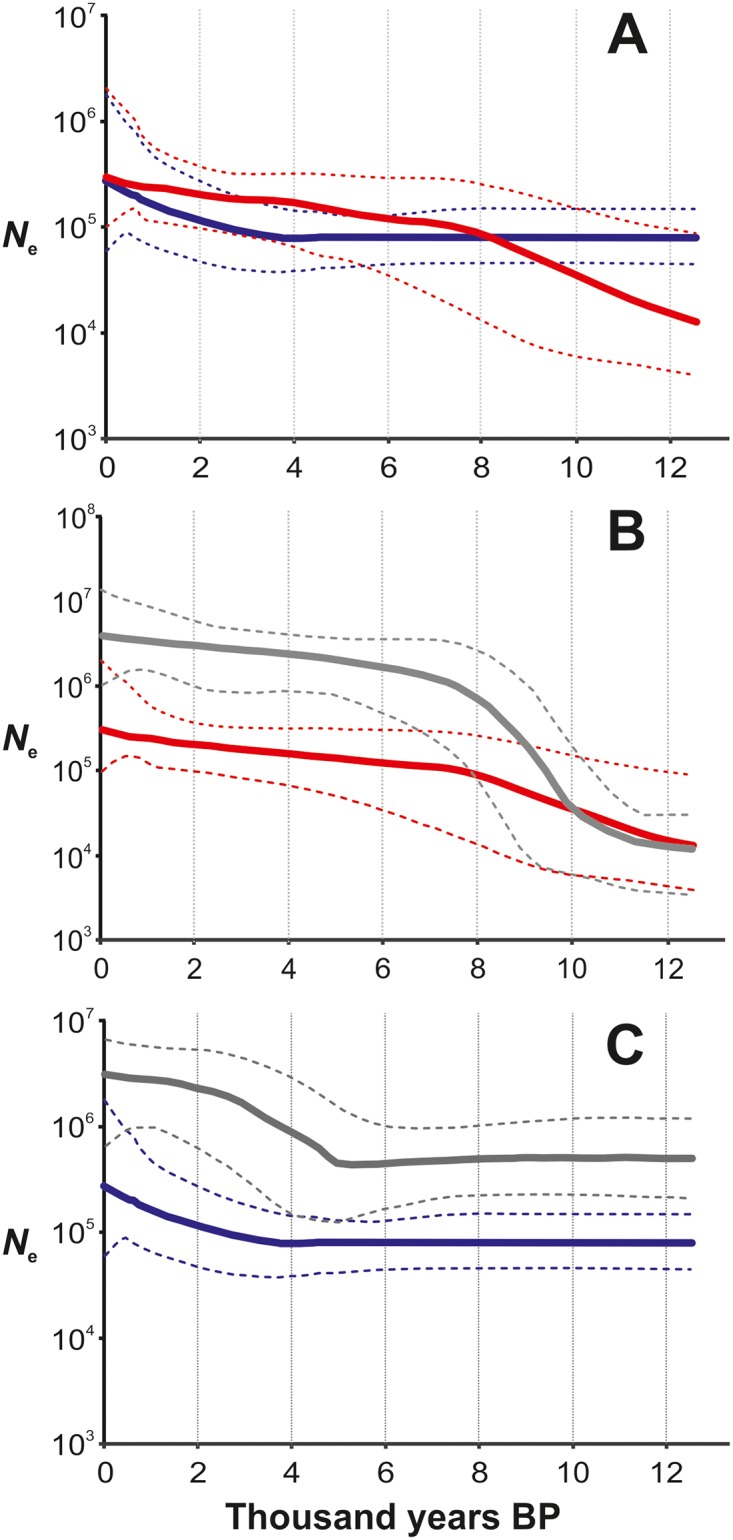
Bayesian Skyline Plots for mtDNA haplogroups H and U in Finland, with European reference data from Fu et al. 2012. The hatched lines denote 95% confidence intervals. A: MtDNA haplogroups H (red) and U (blue) in Finland. B: Haplogroup H in Finland (red) and in Europe (grey). C: Haplogroup U in Finland (blue) and in Europe (grey).

The Bayesian skyline plots show substantially smaller effective population sizes for haplogroups in the HUNT than in the FARM group in Finland, as well as for individual haplogroups in these groups (Fig 4). The FARM haplogroups do show a relatively early population growth: assuming a mutation frequency of 1.69 x 10^-8^ substitutions^-1^ site^-1^ year^-1^ this occurred c. 9 kya, slightly later than estimated in e.g. [32, 38] for Western European H haplotypes. However, the HUNT group BSPs do show a population growth, but relatively late c. 4 kya. In relative terms, the approximate start of population growth in the HUNT haplogroups occur at 0.2 times the FARM group growth start time.

### Y-chromosomes

Y-chromosomal STR haplotype and haplogroup data was obtained for altogether 584 Finnish males. Among this data 294 unique Y-STR haplotypes were observed corresponding an overall haplotype diversity *Ĥ =* 0.9863 ± 0.0019 (SD) (Table 2). Ninety-one percent of the samples fell into haplogroups N1c1 (*N* = 289) and I1 (*N* = 242).

**Table 2:** Y-chromosomal haplogroup frequencies and basic diversity indices. Note that 35 haplotypes for which the sampling location in Finland was unknown are included in “Finland”.

Hg	FINLAND				SOUTHWEST				NORTHEAST			
	*N*	*f*	*A*	*Ĥ*	*N*	*f*	*A*	*Ĥ*	*N*	*f*	*A*	*Ĥ*
**N1c**	289	0.495	147	0.966 ± 0.007	115	0.376	71	0.978 ± 0.006	162	0.667	84	0.947 ± 0.013
**I1**	242	0.414	106	0.970 ± 0.005	170	0.556	84	0.967 ± 0.007	58	0.239	33	0.959 ± 0.014
**I2**	10	0.017	10	1.000 ± 0.045	2	0.007	2	1.000 ± 0.500	6	0.025	6	1.000 ± 0.096
**R1a**	22	0.038	16	0.931 ± 0.046	11	0.036	11	1.000 ± 0.039	9	0.037	3	0.556 ± 0.165
**Q**	1	0.002	1	1	1	0.003	1	1	0	0.000	0	0
**R1b**	16	0.027	10	0.867 ± 0.079	6	0.020	5	0.933 ± 0.122	8	0.033	3	0.464 ± 0.200
**E1b1**	3	0.005	3	1.000 ± 0.272	1	0.003	1	1	0	0.000	0	0
**J**	1	0.002	1	1	0	0.000	0	0	0	0.000	0	0
***ALL***	***584***	***1*.*000***	***294***	***0*.*986 ± 0*.*002***	***306***	***0*.*524***	***175***	***0*.*987 ± 0*.*002***	***243***	***0*.*416***	***129***	***0*.*973 ± 0*.*006***

Hg = haplogroup, *N* = number of samples, *f* = haplogroup frequency, *A* = number of unique haplotypes, *Ĥ* = within-Hg haplotype diversity.

**Fig 5:**
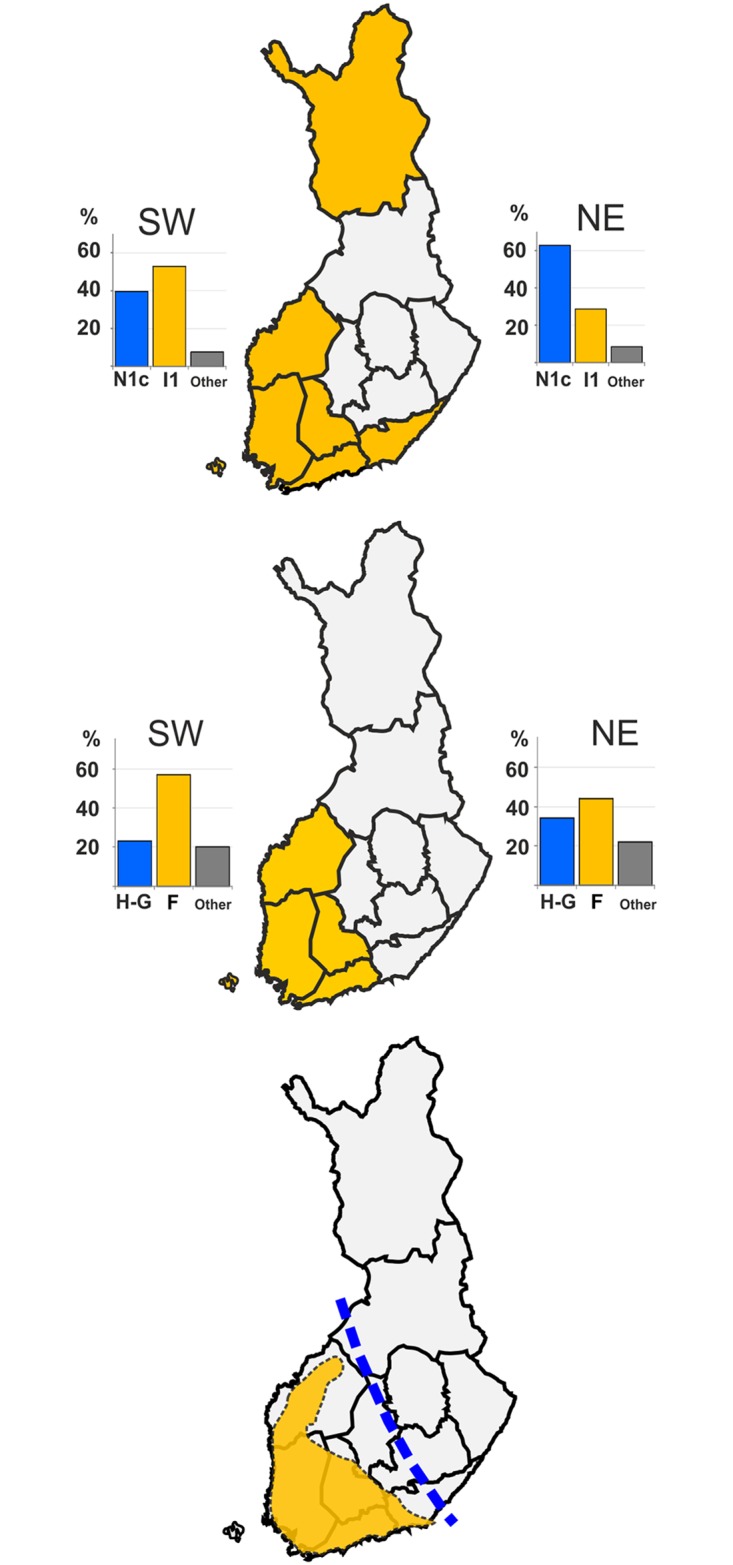
Spatial patterns in different marker classes and in archaeological evidence for Combed Ware Culture in Finland. A: Division maximizing Y-STR haplotype differences, and frequencies of the main Y-haplogroups in Finland. B: Division maximizing the difference between Hunter-Gatherer (H-G: hgs U & V) and Farmer (F: hgs H, T, J & K) mtDNA haplogroups and their frequencies. C: The extent of Corded-Ware Culture (CWC; data from www.nba.fi) in Finland, and the approximate location of the first political border between Sweden and Novgorod (AD 1323; hatched blue line).

The samples were assigned to regions NE and SW, which were defined based on haplotype information in Palo et al. 2009 [23]. Regional haplogroup frequencies are consistent with previous studies [22], with N-frequencies highest in eastern subpopulations, and decreasing moving West. The opposite pattern is seen in the I-haplogroup; high frequency on the western coast and low in the east. The ratio of N1c1/I1 frequencies show strikingly similar spatial pattern with the ratio of mtDNA HUNT/FARM haplogroup frequencies (Fig 5).

The overall haplogroup distribution disparity is reflected in the differentiation estimates: considering the N1c1 and I1 data (representing 91% of the total data), significant differentiation between the NE and SW was observed on the allelic (*Φ*_ST_ = 0.107), but not on the haplotypic level (*F*_ST_ = 0.010). Nevertheless, no significant differences were observed in the diversity between the regions NE and SW (*Ĥ* = 0.9733 ± 0.0061 vs. *Ĥ* = 0.9867 ± 0.0024). Within the haplogroups the haplotype diversity was similar as well: N1c1-haplogroup showed 147 (*Ĥ* = 0.9657 ± 0.0067) and I1-haplogroup 106 (*Ĥ* = 0.9699 ± 0.0048) unique haplotypes. No differences in the haplotype diversities within the N- and I-haplogroups were observed in NE (*Ĥ*_N1c1_ = 0.9473 ± 0.0132; *Ĥ*_I_ = 0.9589 ± 0.0139) or in SW (*Ĥ*_N1c1_ = 0.978±0.0062 and *Ĥ*_I_ = 0.9669±0.0069 respectively).

However, there was a clear difference in the number of observed subhaplogroups between the N1c1 and I1. Within these two major clades, most Finnish Y-chromosomes fall into subhaplogroups of N1c1a1a-L1026 and I1a-DF29 (Tables 2 and S3). Of the other observed haplogroups, I1a1b-L22, with an overall frequency of 29% (71% of the I1 haplotypes), is commonly considered the major “Nordic” branch. In the Family Tree data I1a2a1b-Z73 is found mostly in Finland and Northern Scandinavia, and haplogroups found almost exclusively in Finland include I1a1b3a-L287, I1a1b3a1-L258, I1a1b3a1a-L296 and I1a1b4-L300.

Geographically some Finnish terminal haplogroups show some regional association within Finland; I1a2a-Z59, I1a2a1-Z60, and I1a3-Z63, as well as I1a1b3a1a-L296 and I1a1b4-L300 were found in the Southwestern part of the country. However, two of the lineages unique to Finland, I1a1b3a-L287 and I1a1b3a1-L258, could be observed throughout the country.

## Discussion

In contrast to Y-chromosome, mtDNA haplotype-level assessments have until now failed to identify clear geographical differences within Finland (see e.g. [23]). Here a haplogroup-level analysis revealed spatial patterns that are very similar for both uniparental markers (Fig 5). In the mtDNA data, the SW/NE divergence can be accounted to stem from the frequency differences of haplogroups that have been associated with farmers (H,J,T,K; more common in the SW) and with hunter-gatherers [3]. There is not enough ancient DNA data to allow such an association for the Y-chromosomal data, largely due to poorer preservation of Y-chromosomes in archaeological material. However, haplogroup I has probably arrived later in Finland, and can be thus associated with farmers, whereas the opposite is true for the N haplogroup (see below).

The genetic border in Finland, similar in both the mtDNA and Y-chromosomal data, is in its sharpness rather exceptional in Europe, and cannot be explained by any observable migration barriers. Roewer et al. [39] observed a similar Y-chromosomal border in Central Europe, and interpreted this to stem from political events in Europe since the Middle Ages. Instead, here we propose that the Finnish genetic border represents vestiges of an ancient border between two modes of subsistence, farming and hunter-gathering. It is very likely that this signal has been dampened by internal migration especially during the last century, but its survival until the present day speaks for its strength in the past. In what follows we elaborate this from the viewpoint of the two marker classes.

### mtDNA

Overall, the mtDNA haplogroups associated with hunter-gatherers and farmers show opposite frequency trends along a SW-NE axis in Finland. Hunter-associated hgs are more common in Eastern and Northern Finland. This applies to mitochondrial hgs U and V (especially U5b). Haplogroup U5, together with U8, is an old haplogroup that arrived to Europe at least 30 kya [40] and, as of yet, practically all ancient DNA studies have proven its prevalence in pre-Neolithic hunter-gatherers in Europe [3], [41], [42], also in Scandinavia [43], see also [3].

The farmer haplogroups H, J, T and K show, in turn, a significant SW-bias in Finland. This is especially strong in haplogroup J. The absolute majority (85%) of J haplotypes belong to subhaplogroup J1c2. J1 is a relatively young European haplogroup, which has been observed in Neolithic remains [44] and references therein. Specifically, J1c has been reported from 5–5.5. ky old Neolithic remains from Iberian peninsula [6].

In our data, haplogroup H shows conflicting subhaplogroup patterns. H as a whole is a very diverse group, and its evolutionary history is complex. The most common subhaplogroups in Finland, H1 and H2, show opposing SW-NE trends, with H1 increasing towards NE. Although the current haplogroup H diversity in Europe has been associated with Neolithic cultures [38] and is very rare in Northern European hunter-gatherers [41, 43], the age of the basal H1 subhaplogroup has been dated back to Pleistocene/Holocene boundary c. 11 ky [45], similar to H3 and V. It has also, unlike the other H subgroups, shown continuity from the early Neolithic to the present in Europe [38]. According to Achilli et al. H1 spread from the Franco-Cantabrian refugium with post-glacial expansion of hunter-gatherers [45]. Analyzing the data more detailed in a subhaplogroup level could elucidate the patterns even further, but was out of the scope of this paper.

### Y-chromosomes

The focal parent haplogroups N-M231 and I-M170 both have their origins in the Paleolithic era, and both are strongly associated with the hunter-gatherer lifestyle. N-M231 originated in Southeast Asia approximately 20 kya, while its subgroup N1c1-M46 arose 12 kya [46], [47], and may have appeared in eastern Finland as early as the immediate post-glacial era [47] as well as in later waves associated with the Finno-Ugric speakers; the Mesolithic hunter-gatherer Kunda and Comb Ceramic cultures [48]. [22] [49] [50]. In Finland, N1c1 is the most common Y-haplogroup [51] [50] with an overall occurrence of 58% and with highest concentration [70.9%] in Northern Carelia in the east [20].

I-M170 arose in the Balkans approximately 22 kya [52] and the splitting of this parental clade into subhaplogroups I1-M253, I2a2a-P37.2, and I2a1-M223 also occurred on the European continent [53] [52]. The subhaplogroup I1-M253 and its further branch I1a-DF29 are most prominent in the Scandinavian countries and western Finland, with greatest frequency of I1-M253 in central Sweden (52%) [54] [55], ISOGG. In Finland I1 shows highest concentration in the western provinces (40%) and lowest in eastern Finland (19%) [22]. The arrival of later I1 subhaplogroups to Finland seems to coincide temporally with the arrival of domesticated animals, according to osteological evidence [56].

### Signal of ancient genetic border

The haplogroup distribution in both markers implies that the SW-NE difference still retained in the contemporary genetic diversity in Finland represents an ancient edge of Neolithic farmer advance. In Central Europe, the amalgamation of the Neolithic and Mesolithic gene pools has been more thorough, most likely due to longer time and environment more favourable for the immigrating farming technology. This is in line with the recent ancient DNA results, showing that the genome of 7 000-year-old Mesolithic individual from Northern Spain associated closer to present day genomes from Finland than to any other Europeans included in the study [57].

Like in many other European populations, the recent demographic history of Finns entails strong population growth and, especially in the 20th century, internal migration. The fact that vestiges of an ancient boundary are still discernible suggests that this pattern has in the past been very distinct and that it endured to a later date in the North of Europe. Indeed, archaeological evidence alludes to prolonged coexistence of farmer and hunter-gatherer populations in the north [43, 58]. This is probably due to the later transition to farming in NE Europe (e.g. [59]). Considering the Near Eastern origin and spreading along a southeast-northwest axis into Europe [4], the Fennoscandian region has been the “Ultima Thule”, the northern fringe colonized last by the Neolithic farmers. In Northern Europe the Neolithic advance slowed down. There are probably a number of reasons for this (such as time needed for development of locally adapted crops, e.g. [60]), but one important reason is space competition between farmers and the indigenous Mesolithic populations [17]. Space competition is a process restricting colonization, well-known from a post-glacial recolonization of Europe by wide variety of taxa [61] [62].

These processes gain support from the effective population sizes in haplogroups H, representing the Neolithic farmers, and U representing the hunter-gatherers (see e.g. [32], [43], [38]). When compared to the European averages in these groups [32], the Bayesian Skyline Plots show overall similarity but with two exceptions. Firstly, the estimated sizes are an order of magnitude lower in Finland, and lack patterns of rapid growth, which reflects the lower carrying capacities of the northern latitudes. In fact, the U haplogroup effective size remains rather constant throughout most of the post-glacial. Secondly, and perhaps more importantly, the hunter-gatherer haplogroup U *N*_e_ starts to grow only c. 3000 years ago—some 2000 years later than in Europe. As speculated also for Europe, this growth probably denotes the adoption of agricultural technology by the hunter-gatherers either independently or after population admixture. Note that although the absolute values of effective size and time of events can be questioned, the relative difference between results here and in [32] should hold as the same mutation rate has been assumed. Unfortunately, the available complete mtDNA genome data did not allow comparisons between different regions of Finland.

In the North-East of Europe, a short growing season and relatively unproductive soil were not favorable for farming. In the same time, the western edge of the taiga zone offered plenty of game and fish. Thus, unlike in Western Europe, the colonizing Neolithic farming communities remained relatively small, and probably were assimilated to hunter communities rather than vice versa. Gradual admixture between the arriving Neolithic farmers and relatively numerous Mesolithic hunters in a limited area (SW Finland) could plausibly explain the fact that, unlike most other European populations, Finns do not speak an Indo-European language (*cf*. [63]). Note that the Neolithization process has been connected to the spread of Indo-European languages into Western Europe, although the process might have been complex [64, 65].

The region identified with mtDNA and Y-chromosomal data (see also [66]) matches spatially with the extent of archaeological finds associated to the Corded-Ware Culture (CWC) in Finland. The CWC flourished in a wide area south of the Baltic Sea c. 4.9–4.3 kya. The CWC people based their subsistence on pastoralism and sedentary farming and spoke Indo-European languages (see [48] and references therein).

The CWC spread into SW Finland c. 4.5 kya, which temporally coincides with the advent of farming in Finland. The geographical NE edge of the CWC in Finland has been sharp, dividing Finland into two cultural spheres (Halinen P, In: Suomen historian kartasto). While causality is hard to prove directly, the geographical boundary patterns between the genes and culture are strikingly similar, and can also be seen in a number of cultural features, some of which have persisted into modern times [67]. Interestingly, the first political border in Finland, the 1323 AD agreement between Sweden and Novgorod, also roughly followed the CWC NE edge and the genetic boundary identified in Finland. The reasons for the localization of this boundary may be ecological: the soil most amenable to field farming can be found in SW Finland. Also the vegetation zones and length of the thermic growing seasons changes along a SW-NE axis in Finland (for maps see Finnish Meteorological Service http://ilmatieteenlaitos.fi/terminen-kasvukausi, in Finnish).

While there is plenty of circumstantial evidence suggesting that the CWC has had a strong influence in SW Finland, the identity of the indigenous hunters is more enigmatic. Finland was colonized soon after deglaciation, probably by “converging human groups gradually taking over deglaciated territories” [42]. The most prominent culture in Finland at the time of the CWC arrival was however the Comb Ceramic Culture (CCC) that has been dated back to c. 6.0 kya and extended to the whole of Finland. The extent of the CCC, matching with the extent of Finno-Ugric-speaking populations, suggests that they spoke Finno-Ugric language. Despite the use of ceramics, their subsistence was based on hunting and foraging.

The scenario postulated here is to some extent similar to the ‘language replacement’ theory [68], suggesting that the invading Neolithic population assimilated the local Fenno-Ugric language. However, questions arise especially of the role of the Saami of northern Finland, Sweden, Norway and Russia (Lapland), which also show high frequencies of HUNT haplogroups of this study (esp. mtDNA U5b and Y N1c1) but a clearly distinct overall genetic composition. The similarities between the Saami and Finns today stem probably from admixture. [69] have suggested that the Saami have contributed the the Finnish gene pool especially in the regions directly south of Lapland. An alternative intriguing possibility, fitting well with the uniparental marker data, is that the present-day Saami in fact represent a population admixture between the Palaeolithic people that colonized the north of Europe via the Norwegian coastal corridor as early as c. 11 kya, and the Mesolithic Finno-Ugric Comb Ceramic Culture. This would plausibly explain the conflicting Franco-Cantabrian/ Asian genetic signals, especially the high frequencies of mtDNA U5b1 and Y-chromosomal N1c1 in the Saami gene pool [50, 70–72] (S2 and S3 Tables) and the Finno-Ugric language spoken by the Saami. Formal investigation of this question, however, is out of the focus of this article.

In conclusion, the haplogroup-level analysis of mtDNA and Y-chromosomal markers indicates a contemporary genetic boundary that most likely denotes the limes of Neolithic advance. The persistence of these genetic signals complies with archaeological evidence and simulation studies showing the late arrival farmers in the north of Europe, and subsequent extended coexistence of farmers and hunters in this area.

## Supporting Information

S1 File Original template for Fig 1, from (Palo et al. 2009). Palo JU, Ulmanen I, Lukka M, Ellonen P, Sajantila A. Genetic markers and population history: Finland revisited. Eur J Hum Genet. 2009;17:1336–46.
(PDF)

S1 Table Complete Finnish mitochondrial sequences (N = 367) analyzed in the study. (PDF)

S2 Table Mitochondrial regional association and haplogroup classification. Population abbreviations coincide with Finnish counties Mikkeli (MI), Central Finland (CF), Kuopio (KU), Kymenlaakso (KY), Northern Carelia (NC), Oulu (OU), Lappi (LA), Åland (AL), Turku (TU), Häme (HA), Vaasa (VA), Uusimaa (UU) and Larsmå (LMO).
(XLSX)

S3 Table Y-chromosomal regional association, haplogroup classification, and Y-haplotypes. Samples designated SG and L are in-house data, while FT samples have been obtained from the Family Tree website (www.familytreedna.com). Identification numbers for FT samples are taken from the individual Family Tree testing kit number. Population abbreviations are congruent to those in mitochondrial Table S2. The abbreviation “ht” refers to samples in which the haplogroup has been predicted from the haplotype.
(XLSX)

S4 Table Main haplogroup frequencies in Finland estimated from complete mtDNA and HV1+2 data sets. (PDF)
