# Supplementary material for: Vestiges of an Ancient Border in the Contemporary Genetic Diversity of North-Eastern Europe
Source: PLoS One. 2015 Jul 1;10(7):e0130331. doi: 10.1371/journal.pone.0130331 (PMC4488853; doi:10.1371/journal.pone.0130331)
Supplement: S1 File — Palo JU, Ulmanen I, Lukka M, Ellonen P, Sajantila A. Genetic markers and population history: Finland revisited. Eur J Hum Genet. 2009;17:1336–46. (PDF) [file pone.0130331.s001.pdf]

European Journal of Human Genetics (2009) 17, 1336–1346
© 2009 Macmillan Publishers Limited All rights reserved 1018-4813/09 $32.00
www.nature.com/ejhg

ARTICLE

Genetic markers and population history: Finland revisited

Jukka U Palo*[,1], Ismo Ulmanen[2], Matti Lukka[2], Pekka Ellonen[3] and Antti Sajantila[1]

[1]*Department of Forensic Medicine, University of Helsinki, Helsinki, Finland;* [2]*Forensic Genetic Paternity Testing Unit, National Institute of Health and Welfare, Helsinki, Finland;* [3]*Department of Molecular Medicine, National Institute of Health and Welfare, Helsinki, Finland*

The Finnish population in Northern Europe has been a target of extensive genetic studies during the last decades. The population is considered as a homogeneous isolate, well suited for gene mapping studies because of its reduced diversity and homogeneity. However, several studies have shown substantial differences between the eastern and western parts of the country, especially in the male-mediated Y chromosome. This divergence is evident in non-neutral genetic variation also and it is usually explained to stem from founder effects occurring in the settlement of eastern Finland as late as in the 16th century. Here, we have reassessed this population historical scenario using Y-chromosomal, mitochondrial and autosomal markers and geographical sampling covering entire Finland. The obtained results suggest substantial Scandinavian gene flow into south-western, but not into the eastern, Finland. Male-biased Scandinavian gene flow into the south-western parts of the country would plausibly explain the large inter-regional differences observed in the Y-chromosome, and the relative homogeneity in the mitochondrial and autosomal data. On the basis of these results, we suggest that the expression of 'Finnish Disease Heritage' illnesses, more common in the eastern/north-eastern Finland, stems from long-term drift, rather than from relatively recent founder effects.
European Journal of Human Genetics (2009) **17,** 1336–1346; doi:10.1038/ejhg.2009.53; published online 15 April 2009

Keywords: Finns; Y-STR; autosomal STR; mtDNA; population history; Finnish Disease Heritage

Introduction

As an isolated outlier population on the European genetic landscape,[1] the Finns have attracted a great deal of interest among the geneticists. Owing to advantageous features in its genetic architecture, for example, overall homogeneity, reduced diversity and increased linkage disequilibrium, the Finnish population is considered as a promising target for gene mapping studies.[2,3] Recent analyses have, nevertheless, suggested a substantial geographical structure in the genetic diversity of the Finns.[4–7]

*Correspondence: Dr JU Palo, Department of Forensic Medicine, PO BOX 40, University of Helsinki, FIN-00014 Helsinki, Finland.
Tel: +358 9 191 27471; Fax: +358 9 191 27518;
E-mail: jukka.palo@helsinki.fi
Received 28 October 2008; revised 11 February 2009; accepted 26 February 2009; published online 15 April 2009

The uniqueness of the Finnish genetic architecture has been explained by a series of founder effects and a subsequent drift in local subisolates. The initial founder effects are generally associated with two colonization waves c. 4000 BP (before present) and 2000 BP to southern and western Finland, commonly referred to as the 'Early settlement area' (*ESA*) (Figure 1).[3,8] Another decisive factor shaping the Finnish gene pool is, allegedly, the peopling of the northern/eastern Finland ('Late settlement area', *LSA*) in the 15th–16th century by small family groups from the Early settlement area of southern Finland.[8] The increase of autozygosity associated with these founder effects is deemed to lie behind the occurrence of the 'Finnish Disease Heritage' (FDH), more than 35 recessive monogenic illnesses more common especially in eastern Finland.[8–10]

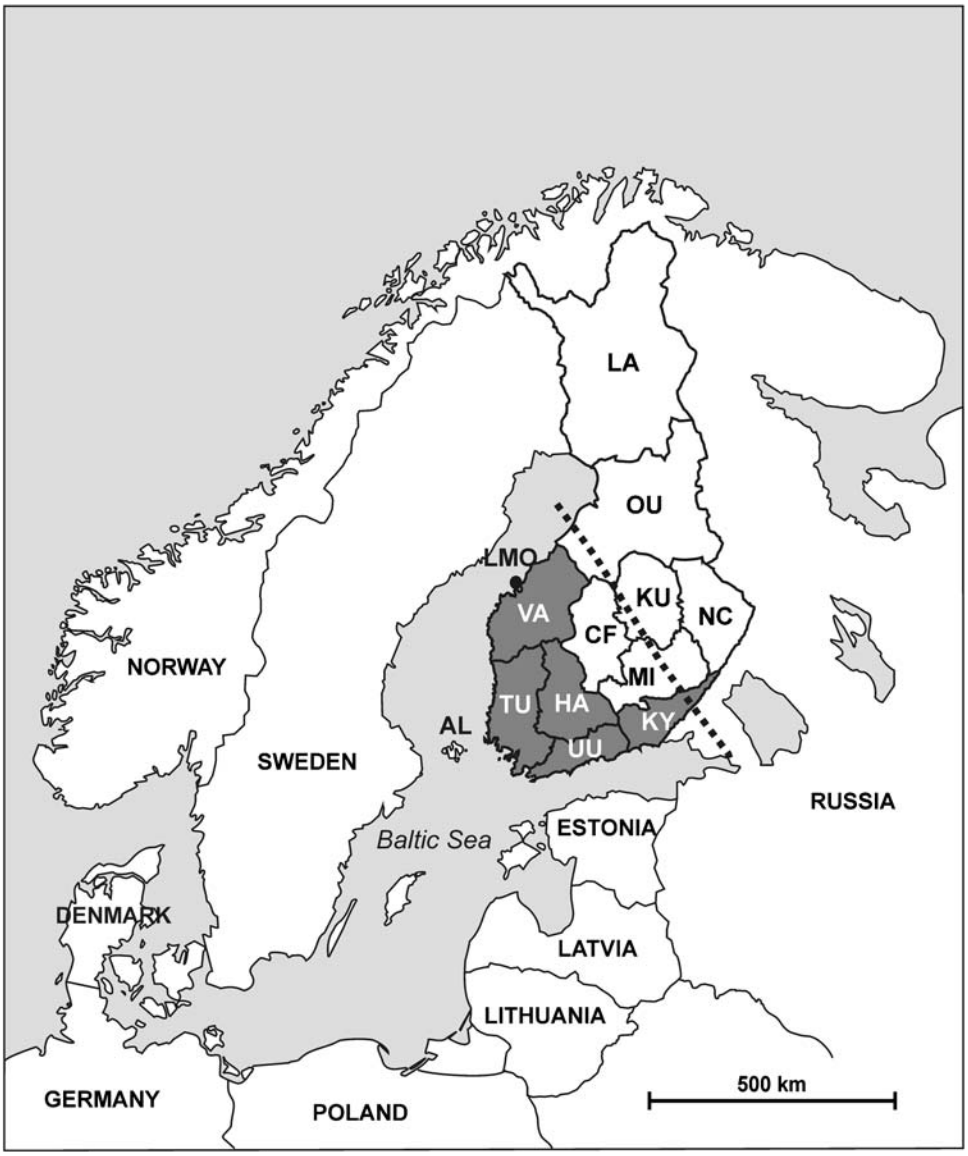

Figure 1 The map of Northern Europe and Finland showing the assessed sub-populations. The dark grey areas show the *a priori* assumed Early settlement area and the hatched line depicts the approximate position of the first political border between Sweden and Novgorod (Russia, year 1323).

If the scenario described above holds, the founder effects and drift in local subisolates should have left distinctive signatures in putatively neutral marker gene diversity as well. In brief, the Finns should show less genetic diversity than other European populations; this reduction should be more drastic in the *LSA*, and the diversity in the *LSA* should represent a subset of the variants observed in the source that is, the *ESA*. In particular, the uniparental mitochondrial DNA (mtDNA) and Y-chromosomal markers should reflect the history more accurately because of their lower molecular effective population size. The uniparental markers should also have similar distribution of diversity, unless there are sex-specific differences in the past demography.

Several studies have indeed shown the *LSA* of eastern/northern Finland to harbour less (neutral) genetic diversity especially in the male-mediated Y chromosome.[11,12] Furthermore, differences in the Y-chromosomal and autosomal variation between western and eastern parts of the country have been revealed.[4,5,7,13] Analyses of linkage disequilibrium are also in line with the expectations based on the demographic history scenario described above.[14,15]

Curiously, however, the mtDNA diversity patterns found among Finns appear to be at odds with the Y-chromosomal variation and also with the proposed ‘medical genetic’ scenario of population history. The Finnish mtDNA pool is shown to harbour levels of diversity comparable with

other European populations, and no significant regional differences have been observed thus far.[16] The observed differences between marker classes in Finland are intriguing. This is especially so, if the patterns are supposed to stem at least partly from a recent event, the internal migration on the 16th century, *i.e.* roughly 15–20 generations ago. Although the Y-chromosomal variation is frequently shown to be geographically more structured,[17–19] the maintenance of mtDNA homogeneity in Finland would require effective female migration between all regions. This is at odds with the subisolate structure leading to an increase of autozygosity and expression of the FDH.

The controversy raises an interesting question: Does the current neutral DNA diversity support the scenario invoked to explain the medical genetic findings? Here we have analysed the genetic differences between 12 different provinces in Finland using Y-chromosomal, mtDNA and autosomal microsatellite data. The mtDNA and autosomal microsatellite data are contrasted with the Y-chromosomal data to examine the history of different regions of Finland, and the regional Finnish diversity is then contrasted with results from several European reference populations. The following basic questions are asked:

(1) Do the regional diversity patterns of Y-chromosomal, mtDNA and autosomal markers deviate from each other?
(2) Do the different sub-populations in Finland have differing affinities to the neighbouring populations?
(3) Are the diversity patterns in all marker classes plausibly explained by the prevailing concept of Finnish population history, that is with bottlenecks associated with the external and internal migration and subsequent drift in local subisolates?

Many aspects of these questions have been earlier touched by a number of studies, but to our knowledge, this is the first study specifically targeting these questions with three classes of neutral markers and geographically structured sampling covering the whole of Finland. On the basis of the obtained results, we question the previous population historical scenarios, which emphasize late founder effects as a main factor behind FDH occurrence and genetic differences within Finland. Instead, we propose an alternative model that accentuates long-term drift in eastern Finland and dissimilar patterns of gene flow into western and eastern parts of Finland.

Materials and methods

Samples and laboratory methods

Altogether, 1126 Finnish males were analysed in this study. These samples were obtained either through paternity testing conducted at the Finnish National Public Health Institute ($N=606$) or collected by the authors (JUP and AS) with informed consents. The Finnish samples also include mtDNA sequences ($N=200$) published earlier.[16]

Subsets of all samples were genotyped with 17 Y-chromosomal (Y-STR) and 9 autosomal microsatellite (aSTR) markers, and a total of 639 bp of mitochondrial hypervariable segment (HVS-)I and II sequence data were obtained. The final data set consisted of altogether 907 Y-STR, 832 mtDNA and 805 autosomal microsatellite profiles, with an actual overlap between marker sets of 58% (Y-STR–mtDNA), 75% (Y-STR and aSTR) and 54% (mtDNA–aSTR). The sample sizes are shown in Table 1.

Table 1 Basic statistics for the assessed markers

	Y-STR				*mtDNA*				*aSTR*				
	N	*A*	*AR*	$\hat{H}$	*N*	*A*	*AR*	$\hat{H}$	*N*	*A*	*AR*	$\hat{H}$	F_{IS}
HA Häme	60	54	35.3	0.996 ± 0.004	81	66	35.5	0.993 ± 0.004	53	6.8	6.6	0.760 ± 0.092	0.038^{NS}
KY Kymi	52	43	32.1	0.982 ± 0.012	74	62	35.7	0.994 ± 0.004	43	6.6	6.5	0.764 ± 0.074	0.024^{NS}
LMO Larsmo	82	56	29.9	0.984 ± 0.005	73	41	26.6	0.974 ± 0.007	74	7.3	6.8	0.734 ± 0.082	0.053^{NS}
TU Turku	56	56	38.0	1.000 ± 0.003	110	91	36.5	0.995 ± 0.003	49	6.8	6.6	0.767 ± 0.072	0.004^{NS}
UU Uusimaa	177	140	34.3	0.991 ± 0.003	64	55	36.3	0.996 ± 0.004	93	7.1	6.5	0.759 ± 0.072	−0.002^{NS}
VA Vaasa	87	71	33.9	0.993 ± 0.004	49	45	37.1	0.996 ± 0.006	78	7.6	7.0	0.764 ± 0.082	−0.023^{NS}
Early settlement	*514*	*354*		*0.994 ± 0.004*	*451*	*265*		*0.995 ± 0.001*	*390*	*8.6*		*0.759 ± 0.084*	*0.015^{NS}*
CF Central Finland	56	47	33.0	0.990 ± 0.007	64	51	33.9	0.991 ± 0.005	49	6.6	6.4	0.765 ± 0.081	0.019^{NS}
KU Kuopio	52	42	31.4	0.985 ± 0.009	89	59	31.6	0.986 ± 0.005	48	6.6	6.4	0.773 ± 0.052	0.000^{NS}
LA Lappi	91	71	33.4	0.992 ± 0.003	52	41	32.6	0.987 ± 0.007	106	7.3	6.7	0.750 ± 0.082	0.034^{NS}
MI Mikkeli	39	32	31.0	0.987 ± 0.010	41	36	35.0	0.993 ± 0.008	42	6.3	6.3	0.767 ± 0.065	−0.060^{NS}
NC Northern Carelia	48	40	32.0	0.983 ± 0.011	47	35	30.3	0.980 ± 0.011	40	6.8	6.8	0.772 ± 0.052	0.008^{NS}
OU Oulu	93	65	30.8	0.983 ± 0.007	75	57	34.1	0.992 ± 0.004	116	7.3	6.5	0.762 ± 0.069	−0.018^{NS}
Late settlement	*379*	*229*		*0.988 ± 0.003*	*368*	*186*		*0.989 ± 0.004*	*402*	*8.3*		*0.764 ± 0.072*	*0.003^{NS}*
AL Åland	14	14	ND	1.000 ± 0.027	13	12	ND	0.987 ± 0.035	13	5.9	ND	0.765 ± 0.062	−0.027^{NS}
**All combined**	**907**	**528**	—	**0.992 ± 0.001**	**832**	**384**	—	**0.993 ± 0.001**	**805**	**9.9**	—	**0.762 ± 0.072**	**0.003^{NS}**

A = number of haplotypes/alleles; AR = haplotype/allelic richness; $\hat{H}$ = haplotype diversity; *N* = number of samples; NS, not significant; ND, not determined.

Haplotypes of 17 Y-chromosomal STR loci were obtained using the AmpF*l*STR Yfiler kit (Applied Biosystems) as described in Palo *et al*.[20] For the data analyses the repeat number of DYS389I was subtracted from that of DYS389II. Multilocus profiles of nine autosomal STR loci (D3S1358, vWA, FGA, TH01, TPOX, CSF1PO, D5S818, D13S317 and D7S820) and Amelogenin were genotyped using the AmpF*l*STR Profiler kit (Promega). All STR products were analysed on an ABI Prism 310 automated sequencer and GeneMapper v. 3.2 software (Applied Biosystems). Concatenated mitochondrial HVS-I and HVS-II sequence data (sites 16 024–16 385 and 72–340, aligned length 639 bp) were obtained following Hedman *et al*.[16]

For the data analyses, the samples were assigned, according to the donor's reported place of residence, to 13 sub-populations (Figure 1; Table 1): Åland (AL), Turku (TU), Uusimaa (UU), Häme (HA), Vaasa (VA), Larsmo (LMO), Kymi (KY), Central Finland (CF), Mikkeli (MI), Kuopio (KU), Northern Carelia (NC), Oulu (OU) and Lapland (LA). These sub-populations correspond to the former Finnish provinces, except LMO, which is a part of the Vaasa province. This locality was included separately in the study as it is almost exclusively a Swedish-speaking community. Roughly 6% of present-day Finns represent the Swedish-speaking minority.

Reference data For the Y-STR and mtDNA comparisons, previously published data from a number of Eurasian populations were included in the analyses. The 7-locus Y-STR data for 44 population samples[21] were obtained through the Y-chromosome Haplotype Reference Database (YHRD).[22] For the analysis, the populations with pairwise Φ_{ST} estimates not differing significantly at the 0.1% level were combined (see Figure 2 in Roewer *et al*[21]), resulting in 22 metasamples. In addition, Swedish population data for 11 Y-STR loci (DYS19, DYS385a.b, DYS389I, DYS389II, DYS390, DYS391, DYS392, DYS393, DYS438 and DYS439) were kindly provided by A. Karlsson.[23]

A subset of the available mtDNA data was chosen to represent different parts of Europe. Data (361 bp HVS-I data; sites 16 024–16 385) were obtained for the following populations: Sweden ($N = 296$, kindly provided by A. Karlsson), Norway ($N = 74$),[24] Estonia ($N = 48$),[25] France ($N = 50$),[26] Russia ($N = 174$),[27,28] Germany ($N = 200$),[29] Italy ($N = 49$),[30] Austria ($N = 101$)[31] and England ($N = 100$).[32]

Statistical analyses

Genetic diversity Y-STR and mtDNA diversity was assessed by calculating the number of haplotypes (A) and by estimating the haplotype ($\hat{H}$) and average gene (π) diversities using the software ARLEQUIN 3.11.[33] To compensate the effect of unequal sample sizes, allelic (haplotype) richness (AR)[34] was estimated in each group using the software CONTRIB 1.02.[35] Unless otherwise mentioned, standard errors or statistical significance of the various

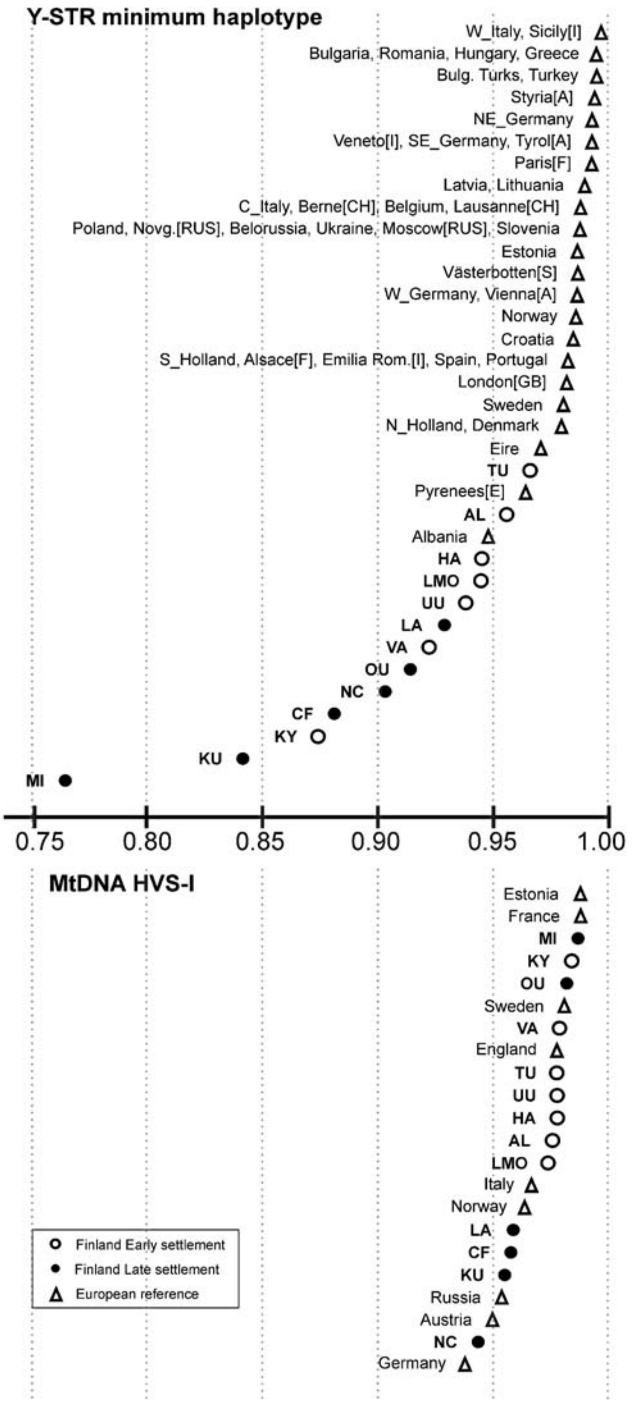

Figure 2 Haplotype diversity point estimates in the Finnish (open and filled circles) and the European reference samples (open triangles), in a descending order. Above the axis: Y-STR (7 loci); below the axis: mtDNA HVS-I.

estimates was obtained through randomization procedures (10 000 steps). Analyses of the autosomal STR data were performed using FSTAT v.2.9.3.[36] Genetic diversity was

estimated by calculating AR and unbiased estimates of gene diversity (*Hs*).[37] Deviations from Hardy–Weinberg equilibrium within sub-populations, sub-population groups and total population were examined by estimating F_{IS} over all loci. Differences in average intrapopulation allelic richness and gene diversity were compared between the sub-population groups using the two-sided test and randomization procedure to assess the statistical significance.

Sub-population differentiation For the subsequent analyses, the sub-population AL was excluded because of its small size. The mainland sub-populations were initially grouped into regions roughly corresponding to the suggested *ESA* (VA, LMO, TU, UU, HA and KY) and *LSA* (MI, CF, KU, NC, OU and LA).

Differences between the sub-populations and sub-population groups were assessed by $\Phi_{ST,}$ for the haplotypic data, and by the analogous $F_{ST,}$[38] for the autosomal diploid STRs. To account for the mutation rate heterogeneity between mtDNA control region sites, Γ-corrected Tamura-Nei substitution model[39] with shape parameter $\alpha=0.2$ was assumed.[40,41] Correlation between genetic and geographical distances between sub-populations was assessed by Mantel tests using the software ARLEQUIN. For the genetic distance matrix, transformed linear $\Phi_{ST}'=\Phi_{ST}/(1-\Phi_{ST})$[42] was used and the geographic distances were given as kilometres separating the major towns in each province (sub-population).

The differentiation among the Finnish sub-populations in each marker class was visualized by simple UPGMA trees, constructed based on the Φ_{ST} distances using program MEGA 4.0.[43] Arguably, dichotomous trees are not optimal means for representing distances between multiple populations. Here, however, UPGMA was chosen because the method allows the visualization of differences between marker sets in a way that, for instance, multidimensional scaling does not.

Analysis of molecular variance (AMOVA)[44] was run first assuming the *ESA*/*LSA* structure described above. AMOVA was also performed in an exploratory fashion for several different modes of clustering (see Table 2).

Population affinities For both the Y-STR and mtDNA sequence data, the relationships of the Finnish sub-populations and the reference populations were assessed by estimating pairwise Φ_{ST} values as described above. The linearized Φ_{ST} distances among populations were visualized by multidimensional scaling, constructed using the ALSCAL procedure in SPSS v. 16.0 (SPSS Inc.).

The relative contribution of neighbouring populations into the Finnish sub-populations was assessed using ADMIX 2.0.[45] With both the Y-STR and mtDNA data, the primarily eastern Finnish sub-populations formed genetically unique clusters, which were assumed as a parental population. However, the composition of these clusters differed between markers. For Y-STR, the admixture was assessed in sub-populations TU, UU, VA, LMO, HA and LA. The eastern Finnish sub-population cluster, CF, KU, MI, NC and OU ($N=288$), was assumed as one parental population. The first set of Y-STR analyses was based on 11-locus haplotypes (see above) and included only the eastern Finland and Sweden as parental populations. The second set included 7-locus haplotypes deposited in the YHRD and assumed the Swedish and the Baltic (Latvia-Lithuania) as parental populations. For the mtDNA data, the admixture proportions were estimated for sub-populations UU, HA, VA, KU and MI. The Finnish parental population was formed by grouping data from CF, NC, OU, LA and TU. Again Sweden was assumed as the other parental population.

In addition, the admixture proportions were estimated for the pooled Finnish *ESA* and *LSA* sub-populations, assuming Swedish and Russian data as parental populations. Here, the *ESA* and *LSA* grouping refers to the sub-population clusters defined above (ESA_Y: TU, UU, VA, LMO, HA and LA; LSA_Y: CF, KU, MI, NC and OU; ESA_{MT}: UU, HA, VA, KU and MI; LSA_{MT}: CF, NC, OU, LA, TU). The analyses were based on 7-locus Y-STR and HVS-I mtDNA data.

Table 2 AMOVA designs and results

	ESA/LSA	*Y1/2/3/4*	*MT1/2/3/4*
F_{CT} (P)			
YSTR	0.012^{NS}	0.047***	0.033**
mtDNA	0.004*	0.000^{NS}	0.011***
aSTR	0.000^{NS}	0.001^{NS}	0.002*
F_{SC} (P)			
YSTR	0.033***	0.002^{NS}	0.013^{NS}
mtDNA	0.003*	0.005**	−0.002^{NS}
aSTR	0.003***	0.002*	0.002*
F_{ST} (P)			
YSTR	0.045***	0.048***	0.046***
mtDNA	0.008***	0.005***	0.009***
aSTR	0.003***	0.003***	0.003***

ESA/LSA: (VA, TU, UU, HA, KY, LMO)/(CF, KU, MI, NC, OU, LA).
Y1/2/3/4: (VA, LMO)/(HA, TU, UU, LA)/(CF, KU, MI, NC, OU)/(KY).
MT1/2/3/4: (VA, UU, HA)/(TU, CF, KU, NC, KY, OU, LA)/(KU, MI)/(LMO).
*$P<0.05$, **$P<0.01$, ***$P<0.001$; NS, not significant.

Results

Y-STR data

Altogether, 528 haplotypes were observed among the 907 Finnish samples analysed with 17 Y-STR markers. There were statistically significant differences in the haplotype diversities between sub-populations (Table 1). The Y-STR haplotype diversities in the Finnish sub-populations and in the European reference populations are presented graphically in Figure 2.

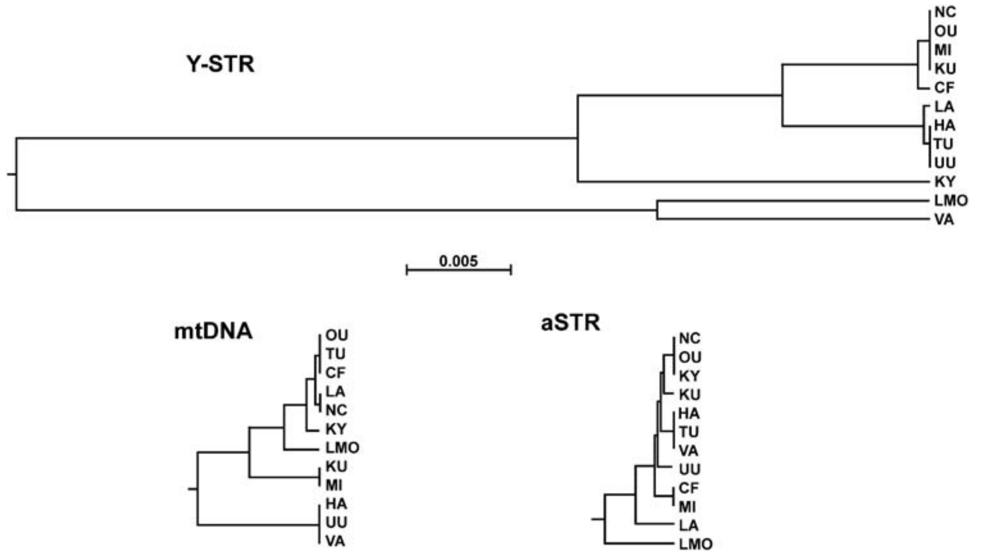

Figure 3 UPGMA clustering of sub-populations based on F_{ST} distances. The trees are drawn in the same scale.

The differentiation estimates between the 12 sub-populations in mainland Finland (AL excluded) ranged from $\Phi_{ST}=0.000$ (14 pairs of populations) to $\Phi_{ST}=0.210$ (KY–LMO). After Bonferroni adjustment,[46] 17 pairwise values out of the 66 comparisons were significantly larger than zero (nominal $P<0.05$, adjusted $P<0.0008$). The Mantel test showed no significant correlation of genetic and geographic distances over all sub-populations ($r=0.109$, $P=0.193$). However, when analysed separately, the correlation was significant among sub-populations within both the *ESA* and *LSA* ($r_{ESA}=0.741$, $P=0.005$; $r_{LSA}=0.719$, $P=0.030$).

Focusing on the *a priori* defined *ESA* ($N=514$) and *LSA* ($N=379$), the southern and western regions of Finland hold significantly more Y-chromosomal diversity ($\hat{H}_{ESA}=0.994\pm0.001$ *vs* $\hat{H}_{LSA}=0.988\pm0.003$; Table 1). AMOVA analysis assuming the *ESA* and *LSA* groups showed notably higher within-region ($F_{SC}=0.033$, $P<0.001$) than among-region variation ($F_{CT}=0.013$). The UPGMA tree (Figure 3) suggests clustering into four groups: a loose group of VA, LMO (*Y1*); the sub-populations TU, UU, HA and LA (*Y2*); the sub-populations NC, OU, MI, KU and CF (*Y3*); and finally KY on its own (*Y4*). In the AMOVA, this grouping renders the within-group variation indistinguishable from zero ($F_{SC}=0.002$, $P=0.194$) and increases the among-group variation ($F_{CT}=0.047$, $P<0.001$).

Mitochondrial DNA

Mitochondrial HVS-I and HVS-II data were obtained for 832 individuals. Altogether, 384 haplotypes were observed, with an estimated haplotype diversity of $\hat{H}=0.993\pm0.001$ and a gene diversity of $\pi=0.012\pm0.006$ in the total data. There were small but significant differences between the mtDNA diversities in different sub-populations (Table 1). The HVS-I haplotype diversities in the Finnish sub-populations and in the European reference populations are presented in Figure 2.

The level of among-sub-population differentiation was substantially lower than that observed in the Y-STR data (arithmetic means Y: $\Phi_{ST}=0.036$, mtDNA: $\Phi_{ST}=0.007$). In the mtDNA, the estimates ranged between $\Phi_{ST}=0$ (17 pairs) to $\Phi_{ST}=0.030$ (KU–VA), with six estimates significant after the Bonferroni correction. No significant correlation was observed between the mtDNA and geographic distances in Finland ($r=0.049$, $P=0.356$), nor within the *ESA* ($r=0.239$, $P=0.199$) or *LSA* ($r=0.051$, $P=0.323$).

As with the Y data, the *ESA* held more mtDNA diversity ($\hat{H}_{ESA}=0.995\pm0.001$ *vs.* $\hat{H}_{LSA}=0.989\pm0.003$). The inter-regional differentiation was also significant, yet lower than that with Y-STRs ($\Phi_{ST}=0.005$, $P<0.001$). AMOVA revealed low, but significant, among-region and within-region differences ($F_{CT}=0.004$, $P=0.002$; $F_{SC}=0.003$, $P<0.050$).

The tree (Figure 3) suggests clustering into four groups, but the compositions differ from those obtained with the Y-STR data: *MT1*: HA, UU and VA; *MT2*: KU and MI; *MT3*: OU, TU, CF, LA, NC and KY and *MT4*: LMO. The *F*-statistics obtained assuming this structure were $F_{CT}=0.011$ ($P<0.001$) and $F_{SC}=-0.002$ ($P=0.906$). Notably, the mtDNA data suggest closer affinity between LMO and the Late settlement area sub-populations than the Y-STR data.

Autosomal microsatellites

In total, 82 alleles were encountered at nine autosomal STR loci genotyped for 805 individuals. The gene diversity over all samples and loci was $Hs=0.762\pm0.072$; the observed

$F_{IS} = 0.006$ did not deviate significantly from zero (95% CI: −0.003 to 0.016; Table 1). Concordant with this, no significant differences in the allelic richness (based on 39 samples) nor in the expected gene diversities between the sub-populations were observed. There were no significant differences in the allele richness or expected heterozygosity between the Early and Late settlement areas, either (AR = 5.63 *vs* 5.56, $P = 0.213$; $Hs = 0.757$ *vs* 0.762, $P = 0.498$).

The genetic differentiation in the autosomal STRs between the 12 sub-populations in Finland is an order of magnitude lower than that in the Y-STR variation. The pairwise values stretch from $F_{ST} = 0$ (18 out of 66 comparisons) to $F_{ST} = 0.015$ (LMO–KU). Only three pairwise F_{ST} estimates, all involving the LMO sub-population, remained statistically significant on the nominal 95% level after the Bonferroni adjustment. However, AMOVA results showed a significant variation among groups within regions ($F_{SC} = 0.0028$, $P < 0.001$), but not among regions ($F_{CT} = 0.0003$, $P < 0.268$).

As with all other markers, the Mantel test revealed no significant correlation between genetic and geographical distances within Finland ($r = 0.131$, $P = 0.216$), nor within *LSA* ($r = -0.022$, $P = 0.505$). In contrast, among the *ESA* sub-populations, a significant correlation was observed ($r = 0.681$, $P = 0.009$).

Population affinities

As within Finland, the distances among the Finnish and the European reference populations were an order of magnitude higher in the Y-STR (average $\Phi_{ST} = 0.129$) than in the mtDNA data (average $\Phi_{ST} = 0.011$). The MDS plots based on the linearized distances are shown in Figure 4. Although the patterns differ depending on the marker type, the Early settlement area sub-populations are generally placed closer to the European references, especially Sweden and Estonia. The KY population, however, is an exception clustering with the eastern sub-populations in the Y-STR (Figure 4a). In case of the LMO sub-population, the Y-chromosomal and mitochondrial data reveal

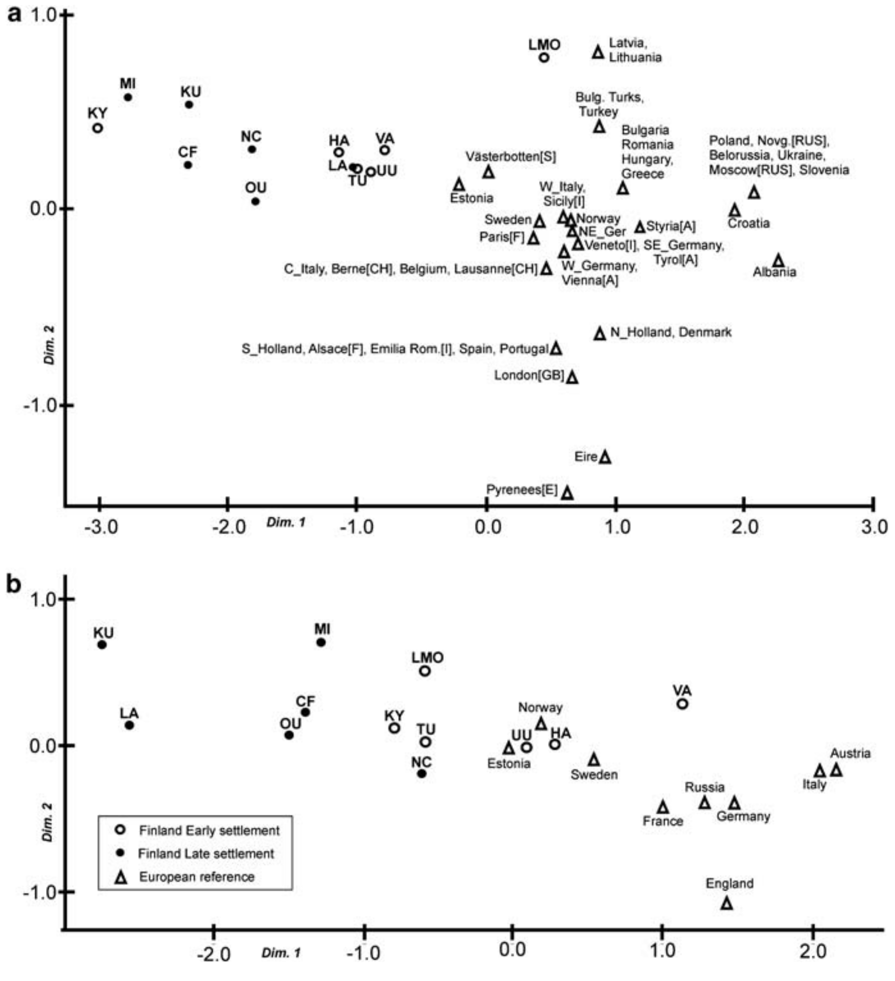

Figure 4 MDS scatterplot based on the linearized Φ_{ST} estimates. (**a**) Seven-locus Y-STR haplotypes. (**b**) mtDNA HVS-I sequence data.

contradictory affinities. The mtDNA data suggest loose clustering among Finnish sub-populations, but the Y-STR data place this sample in the vicinity of the Baltic populations, Latvia and Lithuania, in the MDS. Nevertheless, based on the pairwise Y-chromosomal Φ_{ST} estimates, the LMO sample is clearly closer to Sweden ($\Phi_{ST}=0.020$) than the two Baltic states ($\Phi_{ST}=0.158$).

The male genetic contribution of two parental populations, the *LSA* sub-population cluster and Sweden, was evaluated in the remaining sub-populations VA, LMO, TU, UU, HA, KY and LA. The LA was included here because of its proximity with the *ESA* sub-populations (Figure 3), which altogether show closer Scandinavian affinity (Figure 4). The analysis based on 11-locus Y-STR haplotypes suggests a substantial 20–30% Swedish contribution in most *ESA* sub-populations and LA (Figure 5a). In the LMO, the Swedish contribution exceeds the Finnish. The TU sub-population does show only negligible Swedish contribution, but the analysis with 7-locus haplotype data and three parental populations suggests c. 30% contribution from the Latvia-Lithuania metasample into this region. In all other sub-populations, the Baltic contribution was low (c. 4% in UU) or came up as negative (rest of the sub-populations). The pairwise Φ_{ST} estimates, however, suggest somewhat closer affinity between TU and Sweden ($\Phi_{ST}=0.111$) than between TU and Latvia-Lithuania ($\Phi_{ST}=0.161$).

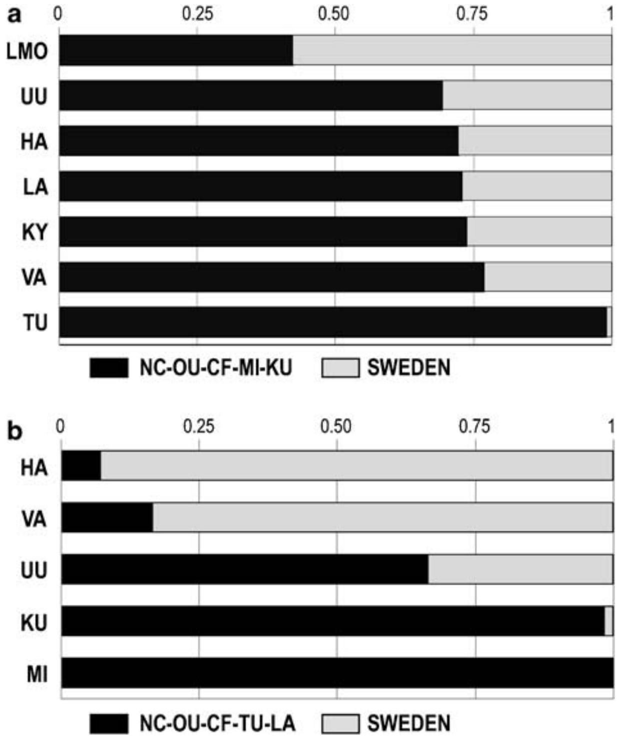

Figure 5 The magnitude of the Scandinavian gene flow in several primarily western Finnish sub-populations estimated from (**a**) the Y-STR data and (**b**) the mtDNA HVS-I + II data.

The admixture analysis of mtDNA haplotypes followed the same logic, although a different clustering was assumed based on the sub-population differentiation. A notable Scandinavian influence was observed in three sub-populations, HA, VA and UU, whereas it was negligible in the KU and MI. These latter two populations are situated in the *a priori* defined *LSA*, despite their intermediate position in the mtDNA tree (Figure 3). However, one must note that the relative uniformity of mtDNA variation in Europe may not allow the identification of the Scandinavian gene flow as clearly as the Y chromosome. The relative contribution of Slavic (Russian) and Swedish populations in both the Y-chromosomal and mtDNA gene pools was estimated separately for the Finnish sub-population clusters. In all analyses, the Slavic contribution came up as negative (data not shown).

Discussion

Recent analyses have convincingly shown the distinctiveness of the Finnish gene pool among the European populations, for example in autosomal SNP markers[1] or in uniparental markers.[47,48] Regional differences in Finland have also been reported.[4–7,12,49] This study, a rather straightforward haplotype-level population genetic analysis, corroborated this picture. However, the degree of segregation and diversity varies between different regions of Finland, as well as between different marker classes; we believe that the observed geographical patterns in the genetic diversity of the uniparental markers have notable corollaries for the population history of Finns.

Different markers – different picture

Compared with the European reference populations, the Y-chromosomal diversity is low, reduced further in the Late settlement area sub-populations and show substantial regional differences. In contrast, the mtDNA diversity does not display marked reduction and shows less, albeit significant, inter-regional variation (Figure 2). The latter observation is at odds with some of the earlier studies.[16] No significant structure was detected in the small 9-locus autosomal STR data within Finland, which at first appears to be in contrast with the recent results from genome-wide SNP data.[7] However, even with 250 000 SNP markers, the differentiation between eastern and western Finland remains low in absolute terms ($F_{ST}=0.0032$)[7] and not drastically different than the estimates obtained here with a small set of autosomal markers.

The markers revealed widely varying differentiation measures between sub-populations and regions. In the Y-STR data, the average differentiation among all sub-populations is c. 10 and 5 times higher than that in the autosomal and mtDNA data (see Figure 3), respectively. The ratio between mtDNA and autosomal STR differentiation is

roughly 3, fitting to the expectations based on the effective (molecular) population sizes.

Gene flow from Scandinavia

In contrast to the findings of Lappalainen *et al*,[4] which in the Y-chromosomal data suggest a clear separation between the Finnish and Swedish gene pools, the admixture analyses suggest substantial Scandinavian contribution that is gene flow into the western and northern parts of the country from the west. In the Y-chromosomal data, the Scandinavian influence was the highest in the coastal sample of Larsmo (LMO) and substantial in most Early settlement area sub-populations as well as in the Lapland (LA; Figure 5). Notably, the Scandinavian gene flow explains the proximity of the northern LA sub-population to the southern and western Finnish sub-populations TU, UU, VA and HA. Unlike the southern parts of Finland, Lapland is not separated from Scandinavia by the Baltic Sea. The regional differences in the Y-chromosomal diversity in Sweden are also small,[23] and gene flow from Sweden could thus homogenize the Y-chromosomal diversity between south-western and northern Finland. The large Y-STR differences between the western and eastern parts of Finland are thus plausibly explained by regionally restricted gene flow, extending to the south-western and northern Finland. The Late settlement area in turn, would seem to retain more of the Fenno-Ugric genetic composition originating from the regions east of Finland. This is reflected in the haplogroup distribution: the Scandinavian haplogroup I occurs with frequencies $>30\%$ only in western Finland.[4] Haplogroup N3, typical for Fenno-Ugric populations of north-eastern Europe,[48,50] is observed in all parts of Finland but reaches high frequencies ($\sim 79\%$) only in eastern Finland. The inter-regional dichotomy results in large Φ_{ST} estimates between eastern and western Finland and, consequently, in the clustering of populations (Figures 3 and 4).

The dissimilarity in the differentiation patterns between Y-chromosomal and mtDNA/autosomal markers, together with the admixture analysis results, further suggests that the Scandinavian gene flow has been male biased. One example of this is the Larsmo (LMO), which shows proximity with the eastern sub-populations in the mtDNA, but strong Scandinavian affinity in the Y-STR data.

The detection of genetic admixture is, obviously, sensitive to the parental populations assumed in the analysis, as well as to the time of the gene flow from the (true) parental populations and subsequent drift. These factors are hard to circumvent and the obtained admixture estimates cannot probably be considered more than qualitatively correct.

Finland revisited

The scenario often invoked to explain the lower genetic diversity, and the prevalence of Finnish disease heritage illnesses especially in the eastern parts of Finland emphasizes founder effects associated with two major immigration waves c. 4000 BP and 2000 BP.[3] This early settlement population has formed a homogeneous source population for the relatively recent inhabitation of the eastern parts of the country.[7,8,51] The patchy occurrence of the FDH in the eastern part of the country is explained by numerous separate founding events from homogeneous source, randomly distributing the disease alleles into the subisolates. The differences between the Early and Late settlement areas thus allegedly stem from this 15th–16th century internal migration.[4,7]

There is in fact little support in the archaeological data for immigration waves into Finland around 4000 BP and 2000 BP. Instead, archaeological evidence suggests that Finland was colonized rather rapidly after the deglaciation c. 10 500 BP.[52–54] The initial colonization has been followed by major immigration waves c. 7500 BP (comb-ceramic culture) and 4500 BP (corded-ware culture). Second, there are no indications that the eastern parts of the country have been uninhabited until the late medieval times as proposed earlier. Model-based approaches predicting the number of inhabitants from the archaeological remains indeed propose a slow overall population growth from c. 10 500 BP onwards (P. Pesonen and M. Tallavaara, unpublished results), although significant reductions of population sizes between c. 6000 BP and 3000 BP in the western and eastern parts of the country are also suggested.

The scenario proposed earlier is also at odds with the current results. First and foremost, it would entail that the *LSA* diversity is a subset of the *ESA* diversity. However, as shown above, the differences especially in the Y-chromosome are not attributable solely to loss of diversity, but show distinctive haplotype and haplogroup distributions, which are more plausibly explained by gene flow into western Finland. The recent bottleneck associated with the settlement of the eastern/northern Finland should have affected both Y-chromosomal and mtDNA diversity relatively similarly. Unlike the Y-chromosomal variation, the mtDNA does not show any sign of reduction and displays fairly homogeneous haplogroup distribution over Finland.[16] The relative uniformity of the mtDNA diversity in Finland has been explained to be because of higher mutation rate in the mtDNA.[11] This appears unlikely. As the mtDNA haplogroups are defined by old mutations and should be rather stable, it is difficult to understand how mutation could produce a haplogroup distribution that is more similar than, for example, that in the Central European populations[55] in only 20 generations. Reversed, the same argument could be used for Y-chromosomal haplogroup distribution: the dichotomous patterns of I and N3 occurrence are not likely to arise because of neutral chance processes such as drift. Therefore, an alternative explanation – male-biased gene flow from Scandinavia to western parts of Finland – appears as more likely in the light of the current results.

The results propose Scandinavian gene flow as a source of inter-regional differences in Finland. As the whole of Finland has been continuously inhabited since the early Holocene,[52] it may be assumed that these early populations have also contributed to the present-day gene pool. It is possible that, at some stage, the majority of the males in this prehistoric population carried the Y chromosomes of haplogroup N3.[48] The subsequent Scandinavian gene flow has then affected the genetic composition of the south-western as well as the northernmost parts of Finland only, creating the large Y-chromosomal differences between western and eastern parts of the country. In other words, the Scandinavian influence can be seen as an additional genetic element in the *ESA* region (and Lapland), whereas the Finno-Ugric genetic component remains still more prominent in the east of Finland. It also fits to the notion of slight yet significant substructure observed in autosomal SNP markers.[7] If this gene flow has been male biased, it could also credibly explain the large differences between the Y-chromosomal and mtDNA/autosomal diversity patterns. Under the model proposed here, the patchy occurrence of FDH diseases, especially in eastern Finland, is better explained by long-term drift, more acute in the sparsely inhabited eastern Finland, rather than by relatively recent founder effects. The distances observed between the *LSA* sub-populations also support high drift in this area (Figure 4). Alleviated by the Scandinavian gene flow, the drift has been less severe in the western parts of the country.

This model is, to our opinion, well supported by other evidence. Already in the prehistoric times, there has been a close tie between south-western Finland, Sweden and Estonia.[56] The south-western parts of Finland were brought under the Swedish rule between c. 1150 and 1300, which led to the emergence of Finland's Swedish-speaking population (today comprising ca. 6%). Another Scandinavian political power, the Danes, were also actively operating along the Baltic coast in the medieval times.[57] Markedly, in the early medieval period, the Swedish reign was not extended further than in the south-western Finland, roughly corresponding to the *ESA*. This has been traditionally explained by the opposing force of Russia (Novgorod); however, ecological factors may also have had an influence on the agricultural population. The current area of south-western Finland, having a distinctive Y-chromosomal variation, correlates conspicuously with the southern boreal ecological zone (thermic growth period maps: http://www.fmi.fi/saa/tilastot_72.html#7) and with the first political border between Sweden and Novgorod (year 1323, Figure 1).

The model for the settling of Finland proposed earlier emphasizes founder effects, both in the initial phases and during the internal immigration in the historical era. On the basis of the current results and other evidence, we have proposed an alternate model that emphasizes long-term drift and gene flow as factors behind the regional differences. The fact that many and grave objections may be advanced against this model of Finnish population history cannot be denied. Nevertheless, the model proposed presented here most plausibly explains the genetic differences observed between the western and eastern parts of Finland and also offers justification for the dissimilarities observed between the marker classes.

Acknowledgements

We thank the sample donors, as well as Dr Bjarne Udd for the Larsmo samples and Andreas Tillman (Karlsson) for the Swedish Y-STR and mtDNA data. Ms Airi Sinkko, Ms Kirsti Höök and Ms Eve Karvinen are thanked for technical assistance. We also thank Dr Ville Pimenoff for many fruitful discussions and two anonymous referees for their helpful comments. The study has been financially supported by Academy of Finland (grants 1109265 and 1111713 to JUP) and by the European Science Foundation's EUROCORES programme (Origin of Man, Language and Languages to AS). This study is part of the multidisciplinary ARGEOPOP project (University of Helsinki).

References

1 Lao O, Lu TT, Nothnagel M *et al*: Correlation between genetic and geographic structure in Europe. *Curr Biol* 2008; **18**: 1241–1248.
2 de la Chapelle A, Wright FA: Linkage disequilibrium mapping in isolated populations: the example of Finland revisited. *Proc Natl Acad Sci USA* 1998; **95**: 12416–12423.
3 Peltonen L, Palotie A, Lange K: Use of population isolates for mapping complex traits. *Nat Rev Genet* 2000; **1**: 182–190.
4 Lappalainen T, Koivumäki S, Salmela E *et al*: Regional differences among the Finns: a Y-chromosomal perspective. *Gene* 2006; **376**: 207–215.
5 Palo JU, Hedman M, Ulmanen I, Lukka M, Sajantila A: High degree of Y-chromosomal divergence within Finland – forensic aspects. *Forensic Sci Int Genet* 2007; **1**: 120–124.
6 Hannelius U, Salmela E, Lappalainen T *et al*: Population substructure in Finland and Sweden revealed by the use of spatial coordinates and a small number of unlinked autosomal SNPs. *BMC Genet* 2008; **9**: 54.
7 Salmela E, Lappalainen T, Fransson I *et al*: Genome-wide analysis of single nucleotide polymorphisms uncovers population structure in Northern Europe. *PLoS ONE* 2008; **3**: e3519.
8 Kere J: Human population genetics: lessons from Finland. *Annu Rev Genomics Hum Genet* 2001; **2**: 103–128.
9 Norio R, Nevanlinna HR, Perheentupa J: Hereditary diseases in Finland. *Ann Clin Res* 1973; **5**: 109–141.
10 Norio R: The Finnish Disease Heritage III: the individual diseases. *Hum Genet* 2003; **112**: 470–526.
11 Sajantila A, Salem AH, Savolainen P, Bauer K, Gierig C, Pääbo S: Paternal and maternal DNA lineages reveal a bottleneck in the founding of the Finnish population. *Proc Natl Acad Sci USA* 1996; **93**: 12035–12039.
12 Hedman M, Pimenoff V, Lukka A, Sistonen P, Sajantila A: Analysis of 16 Y STR loci in the Finnish population reveals a local reduction in the diversity of male lineages. *Forensic Sci Int* 2004; **142**: 37–43.
13 Kittles RA, Perola M, Peltonen L *et al*: Dual origins of Finns revealed by Y chromosome haplotype variation. *Am J Hum Genet* 1998; **62**: 1171–1179.
14 Varilo T, Laan M, Hovatta I, Wiebe V, Terwilliger JD, Peltonen L: Linkage disequilibrium in isolated populations: Finland and a young sub-population of Kuusamo. *Eur J Hum Genet* 2000; **8**: 604–612.

15 Service S, DeYoung J, Karayiorgou M *et al*: Magnitude and distribution of linkage disequilibrium in population isolates and implications for genome-wide association studies. *Nat Genet* 2006; **38**: 556–560.
16 Hedman M, Brandstätter A, Pimenoff V *et al*: Finnish mitochondrial DNA HVS-I and HVS-II population data. *Forensic Sci Int* 2007; **172**: 171–178.
17 Seielstad MT, Minch E, Cavalli-Sforza LL: Genetic evidence for a higher female migration rate in humans. *Nat Genet* 1998; **20**: 278–280.
18 Hamilton G, Stoneking M, Excoffier L: Molecular analysis reveals tighter social regulation of immigration in patrilocal populations than in matrilocal populations. *Proc Natl Acad Sci USA* 2005; **102**: 7476–7480.
19 Wilkins JF, Marlowe FW: Sex-biased migration in humans: what should we expect from genetic data? *Bioessays* 2006; **28**: 290–300.
20 Palo JU, Pirttimaa M, Bengs A *et al*: The effect of number of loci on geographical structuring of Y-STR data in Finland. *Int J Legal Med* 2008; **122**: 449–456.
21 Roewer L, Croucher PJP, Willuweit S *et al*: Signature of recent historical events in the European Y-chromosomal STR haplotype distribution. *Hum Genet* 2005; **116**: 279–291.
22 Willuweit S, Roewer L: Y chromosome haplotype reference database: update. *Forensic Sci Int Genet* 2007; **1**: 83–87.
23 Karlsson AO, Wallerström T, Götherström A, Holmlund G: Y-chromosome diversity in Sweden – a long-time perspective. *Eur J Hum Genet* 2006; **14**: 963–970.
24 Passarino G, Cavalleri GL, Lin AA, Cavalli-Sforza LL, Borresen-Dale AL, Underhill PA: Different genetic components in the Norwegian population revealed by the analysis of mtDNA and Y chromosome polymorphisms. *Eur J Hum Genet* 2002; **10**: 521–529.
25 Sajantila A, Lahermo P, Anttinen T *et al*: Genes and languages in Europe – an analysis of mitochondrial lineages. *Genet Res* 1995; **5**: 42–52.
26 Rousselet F, Mangin P: Mitochondrial DNA polymorphisms: a study of 50 French Caucasian individuals and application to forensic casework. *Int J Legal Med* 1998; **111**: 292–298.
27 Orekhov V, Poltoraus A, Zhivotovsky LA, Spitsyn V, Ivanov P, Yankovsky N: Mitochondrial DNA sequence diversity in Russians. *FEBS Lett* 1999; **445**: 197–201.
28 Kornienko I, Vodolazhskii D, Afanaseva G, Ivanova P: Polymorphism of the central region of D-loop of mitochondrial DNA and personality identification by forensic medicine methods. *Sud Med Ekspert* 2004; **47**: 27–32.
29 Lutz S, Weisser HJ, Heizmann J, Pollak S: Location and frequency of polymorphic positions in the mtDNA control region of individuals from Germany. *Int J Legal Med* 1998; **111**: 67–77.
30 Francalacci P, Bertranpetit J, Calafell F, Underhill PA: Sequence diversity of the control region of mitochondrial DNA in tuscany and its implications for the peopling of Europe. *Am J Phys Anthropol* 1996; **100**: 443–460.
31 Parson W, Parsons TJ, Scheithauer R, Holland MM: Population data for 101 Austrian Caucasian mitochondrial DNA d-loop sequences: application of mtDNA sequence analysis to a forensic case. *Int J Legal Med* 1998; **111**: 124–132.
32 Piercy R, Sullivan KM, Benson N, Gill P: The application of mitochondrial-DNA typing to the study of white caucasian genetic identification. *Int J Legal Med* 1993; **106**: 85–90.
33 Excoffier L, Laval LG, Schneider S: Arlequin ver. 3.0: an integrated software package for population genetic data analysis. *Evol Bioinform Online* 2005; **1**: 47–50.
34 Mousadik A, Petit RJ: High level of genetic differentiation for allelic richness among populations of the argan tree [*Argania spinosa* (L.) Skeels] endemic to Morocco. *Theor Appl Genet* 1996; **92**: 832–839.
35 Petit RJ, El Mousadik A, Pons O: Identifying populations for conservation on the basis of genetic markers. *Conserv Biol* 1998; **12**: 844–855.
36 Goudet J: FSTAT, a program to estimate and test gene diversities and fixation indices (version 2.9.3). Available from http://www.unil.ch/izea/softwares/fstat.html, 2001.
37 Nei M: *Molecular Evolutionary Genetics*. New York: Columbia University Press, 1987.
38 Weir BS, Cockerham CC: Estimating *F*-statistics for the analysis of population structure. *Evolution* 1984; **38**: 1358–1370.
39 Tamura K, Nei M: Estimation of the number of nucleotide substitutions in the control region of mitochondrial DNA in humans and chimpanzees. *Mol Biol Evol* 1993; **10**: 512–526.
40 Von Haeseler A, Sajantila A, Paabo S: The genetical archaeology of the human genome. *Nat Genet* 1996; **14**: 135–140.
41 Meyer S, Weiss G, von Haeseler A: Pattern of nucleotide substitution and rate heterogeneity in the hypervariable regions I and II of human mtDNA. *Genetics* 1999; **1523c**: 1103–1110.
42 Slatkin M: A measure of population subdivision based on microsatellite allele frequencies. *Genetics* 1995; **139**: 457–462.
43 Tamura K, Dudley J, Nei M, Kumar S: MEGA4: molecular evolutionary genetics analysis (MEGA) software version 4.0. *Mol Biol Evol* 2007; **24**: 1596–1599.
44 Excoffier L, Smouse PE, Quattro JM: Analysis of molecular variance inferred from metric distances among DNA haplotypes: application to human mitochondrial DNA restriction data. *Genetics* 1992; **131**: 479–491.
45 Dupanloup I, Bertorelle G: Inferring admixture proportions from molecular data: Extension to any number of parental populations. *Mol Biol Evol* 2001; **18**: 672–675.
46 Rice WR: Analyzing tables of statistical tests. *Evolution* 1989; **43**: 223–225.
47 Lappalainen T, Laitinen V, Salmela E *et al*: Migration waves to the Baltic Sea region. *Ann Hum Genet* 2008; **72**: 337–348.
48 Pimenoff VN, Comas D, Palo JU, Vershubsky G, Kozlov A, Sajantila A: Northwest Siberian Khanty and Mansi in the junction of West and East Eurasian gene pools as revealed by uniparental markers. *Eur J Hum Genet* 2008; **16**: 1254–1264.
49 Kittles RA, Bergen AW, Urbanek M *et al*: Autosomal, mitochondrial, and Y chromosome DNA variation in Finland: evidence for a male-specific bottleneck. *Am J Phys Anthropol* 1999; **108**: 381–399.
50 Balanovsky O, Rootsi S, Pshenichnov A *et al*: Two sources of the Russian Patrilineal heritage in their Eurasian context. *Am J Hum Genet* 2008; **82**: 236–250.
51 de la Chapelle A: Disease gene-mapping in isolated human-populations – the example of Finland. *J Med Genet* 1993; **30**: 857–865.
52 Carpelan C: On the postglacial colonization Eastern Fennoscandia; in Huurre M (ed): *Dig It All: Papers Dedicated to Ari Siiriäinen*. Helsinki: The Finnish Antiquarian Society and The Archaeological Society of Finland, 1999, pp 151–172.
53 Nuñez M: Finland's settling model revisited. *Mankind Q* 2002; **43**: 155–175.
54 Bergman I, Olofsson A, Hörnberg G, Zackrisson O, Hellberg E: Deglaciation and colonization: pioneer settlements in Northern Fennoscandia. *J World Prehist* 2004; **18**: 155–177.
55 Torroni A, Huoponen K, Francalacci P *et al*: Classification of European mtDNAs from an analysis of three European populations. *Genetics* 1996; **144**: 1835–1850.
56 Carpelan C: Essay on archaeology and languages in the western end of the Uralic zone; in Nurk Aea (ed): *Congressus Nonus Internationalis Fenno-Ugristarum*. Tartu: Estonia, 2000, Vol. 1, pp 7–38.
57 Jutikkala E, Pirinen K: *History of Finland*. Helsinki: WSOY, 2003.
