# Supplementary material for: Vestiges of an Ancient Border in the Contemporary Genetic Diversity of North-Eastern Europe
Source: PLoS One. 2015 Jul 1;10(7):e0130331. doi: 10.1371/journal.pone.0130331 (PMC4488853; doi:10.1371/journal.pone.0130331)
Supplement: S1 Table — (PDF) [file pone.0130331.s002.pdf]

S1 Table. Complete Finnish mitochondrial sequences (N = 367) analyzed in the study.

GenBank accession no	N	Origin	Reference
**AY339402–AY339593**	192	Central Finland	Finnilä S, Lehtonen MS, Majamaa K (2001) Phylogenetic network for European mtDNA. *AJHG* 68:1475-1484
**JX171078–JX171140**	63	Northern Finland	Soini HK, Moilanen JS, Finnilä S, Majamaa K (2012) Mitochondrial DNA sequence variation in Finnish patients with matrilinear diabetes mellitus. *BioMed Central Research Notes* 5:350–361
**EU753433, EU784076, FJ543390, FJ801039, GQ176284, GU206811, GU391321, GU949563, HM116534, HM856585, HQ022823, HQ658464, HQ840516, JF298814, JF813785, JF813786, JF903928, AY195773*, JF837819****	19	Finland	Greenspan, B. Submitted Family Tree DNA - Genealogy by Genetics, Ltd., 1445 North Loop West, Suite 820, Houston, TX 77008, USA.<br><br>*Mishmar D, Ruiz-Pesini E, Golik P, Macaulay V, Clark AG et al. (2003) Natural selection shaped regional mtDNA variation in humans. Proc. Natl. Acad. Sci. U.S.A. 100: 171-176.<br><br>** Pike DA, Barton TJ, Bauer SL, Kipp E (2010) MtDNA haplogroup T phylogeny based on full mitochondrial sequences. J Genet Geneal 6: 1-24.
**n/a**	93	Finland	1000 Genomes Project Consortium 2012
