# Supplementary material for: Vestiges of an Ancient Border in the Contemporary Genetic Diversity of North-Eastern Europe
Source: PLoS One. 2015 Jul 1;10(7):e0130331. doi: 10.1371/journal.pone.0130331 (PMC4488853; doi:10.1371/journal.pone.0130331)
Supplement: S4 Table — (PDF) [file pone.0130331.s005.pdf]

S4 Table. Main haplogroup frequencies in Finland estimated from complete mtDNA and HV1+2 data sets.

Haplogroup	Complete		HV1+2	
	N	%	N	%
**H**	94	25.6	276	33.2
**>H1**	45	12.3	94	11.3
**HV**	1	0.3	5	0.6
**I**	18	4.9	10	1.2
**J**	28	7.6	46	5.5
**K**	23	6.3	46	5.5
**R**	1	0.3	6	0.7
**T**	20	5.4	51	6.1
**U**	86	23.4	202	24.3
**>U5**	68	18.5	175	21.0
**>>U5a**	20	5.5	53	6.4
**>>U5b**	48	13.1	122	14.7
**V**	40	10.9	30	3.6
**>V1**	17	4.6	9	1.1
**>V7**	9	2.5	14	1.7
**W**	42	11.4	31	3.7
**X**	5	1.4	15	1.8
**Z**	9	2.5	4	0.5
**Others/undefined**	-	-	110	13.2
**Total**	367	100.0	832	100.0
